# Krüppel-like factor 10 modulates stem cell phenotypes of pancreatic adenocarcinoma by transcriptionally regulating notch receptors

**DOI:** 10.1186/s12929-023-00937-z

**Published:** 2023-06-12

**Authors:** Yi-Chih Tsai, Kung Hung Cheng, Shih Sheng Jiang, John R. Hawse, Shun En Chuang, Su Liang Chen, Tze-Sing Huang, Hui-Ju Ch’ang

**Affiliations:** 1 National Institute of Cancer Research, National Health Research Institutes, R1-2034, 35 Keyan Road, Zhunan, Miaoli County, 35053 Taiwan; 2 Institute of Biomedical Sciences, National Sun Yat-Sen University, Kaohsiung, Taiwan; 3 Department of Biochemistry and Molecular Biology, Mayo Clinic, Rochester, MN USA; 4 Program for Cancer Biology and Drug Discovery, College of Medical Science and Technology, Taipei Medical University, Taipei, Taiwan; 5 Department of Oncology, School of Medicine, College of Medicine, National Cheng Kung University, Tainan, Taiwan

**Keywords:** Krüppel-like factor 10, Notch signaling, *Notch-3/4*, *ELF3*, Pancreatic adenocarcinoma

## Abstract

**Background:**

Pancreatic adenocarcinoma (PDAC) is well known for its rapid distant metastasis and local destructive behavior. Loss of Krüppel-like factor 10 (KLF10) contributes to distant migration of PDAC. The role of KLF10 in modulating tumorigenesis and stem cell phenotypes of PDAC is unclear.

**Methods:**

Additional depletion of KLF10 in KC (LSL: Kras^G12D^; Pdx1-Cre) mice, a spontaneous murine PDAC model, was established to evaluate tumorigenesis. Tumor specimens of PDAC patients were immune-stained of KLF10 to correlate with local recurrence after curative resection. Conditional overexpressing KLF10 in MiaPaCa and stably depleting KLF10 in Panc-1 (Panc-1-pLKO-shKLF10) cells were established for evaluating sphere formation, stem cell markers expression and tumor growth. The signal pathways modulated by KLF10 for PDAC stem cell phenotypes were disclosed by microarray analysis and validated by western blot, qRT-PCR, luciferase reporter assay. Candidate targets to reverse PDAC tumor growth were demonstrated in murine model.

**Results:**

KLF10, deficient in two-thirds of 105 patients with resected pancreatic PDAC, was associated with rapid local recurrence and large tumor size. Additional KLF10 depletion in KC mice accelerated progression from pancreatic intraepithelial neoplasia to PDAC. Increased sphere formation, expression of stem cell markers, and tumor growth were observed in Panc-1-pLKO-shKLF10 compared with vector control. Genetically or pharmacologically overexpression of KLF10 reversed the stem cell phenotypes induced by KLF10 depletion. Ingenuity pathway analysis and gene set enrichment analysis showed that Notch signaling molecules, including Notch receptors 3 and 4, were over-expressed in Panc-1-pLKO-shKLF10. KLF10 transcriptionally suppressed *Notch-3* and *-4* by competing with E74-like ETS transcription factor 3, a positive regulator, for promoter binding. Downregulation of Notch signaling, either genetically or pharmacologically, ameliorated the stem cell phenotypes of Panc-1-pLKO-shKLF10. The combination of metformin, which upregulated KLF10 expression via phosphorylating AMPK, and evodiamine, a non-toxic *Notch-3* methylation stimulator, delayed tumor growth of PDAC with KLF10 deficiency in mice without prominent toxicity.

**Conclusions:**

These results demonstrated a novel signaling pathway by which KLF10 modulates stem cell phenotypes in PDAC through transcriptionally regulating Notch signaling pathway. The elevation of KLF10 and suppression of Notch signaling may jointly reduce PDAC tumorigenesis and malignant progression.

**Supplementary Information:**

The online version contains supplementary material available at 10.1186/s12929-023-00937-z.

## Introduction

Pancreatic ductal adenocarcinoma (PDAC) is one of the deadliest malignancies, typically presents with early distant metastasis and local invasion. PDAC is resistant to most cancer therapies, and despite recent progress in early detection and therapeutic efficacy, the overall patient survival rate remains low [1]. Therefore, the development of new biologic markers and therapeutic targets for PDAC is an urgent matter.

Our previous studies have revealed that Krüppel-like factor 10 (KLF10), a zinc finger-containing transcription factor originally identified as an early response gene of TGFβ (TGFβ-inducible early gene-1; TIEG1), was downregulated in two-thirds of PDAC patients due to epigenetic and post-translational regulation [2, 3]. KLF10 was reported to positively respond to an anti-proliferative signal of TGFβ [4]. Previous studies, including our own, have reported that KLF10 deficiency activates the SDF/CXCR4 signaling pathway and transcriptionally upregulates Sirtuin 6 and Slug to modulate glycolysis and epithelial–mesenchymal transition (EMT) for distant metastasis in PDAC and lung cancer [5–7]. In contrast to TGFβ in advanced cancers, loss of KLF10 was associated with rapid distant metastasis, relatively short survival, and resistance to radiotherapy [3, 8]. Distinguishing the anti-proliferative functions from the pro-metastatic functions of TGFβ reveals KLF10 to be a potential target for the development of therapies for cancers with aberrant TGFβ pathways, such as PDAC [6]. However, the effects of KLF10 on the cancer stem cell phenotypes of PDAC are still unclear.

In this study, we found that additional deletion of KLF10 in the murine model of spontaneous pancreatic cancer accelerated PDAC tumorigenesis. KLF10 deletion transcriptionally upregulated Notch signaling molecules to promote sphere formation, and tumorigenesis of PDAC. Mechanistically, KLF10 competed with E74-like ETS transcription factor 3 (ELF3) for promoter binding of *Notch-3* and *-4* for transcription regulation. Upregulating KLF10 and/or suppressing Notch signaling, genetically or pharmacologically, delayed tumor growth of PDAC with KLF10 deficiency.

## Materials and methods

### Cell culture and chemicals

Human pancreatic cancer cell lines, Panc-1 (BCRC 60284) and MiaPaCa (BCRC 60139), were purchased from the Bioresource Collection and Research Center, Taiwan, and were authenticated by DNA fingerprinting. Panc-1 cells labeled with firefly luciferase plasmid vector (Panc-1-Luc) were kindly provided from Dr. Kelvin K. C. Tsai from Taipei Medical University. The mouse primary pancreatic adenocarcinoma (PDAC) cells were cultured in RPMI-1640 medium supplemented with 10% fetal bovine serum, 100 units/mL penicillin, 100 mg/mL streptomycin and 2 mM glutamine at 37 °C in a 5% CO_2_ incubator; maintained for < 6 passages and histo-pathologically characterized through SCID mice xenograft studies. Metformin hydrochloride (D150959,), dorsomorphin (Compound C, P5499) and evodiamine (E3531) were purchased from Sigma-Aldrich. *N*-[*N*-(3,5-Difluorophenacetyl-l-alanyl)]-(*S*)-phenylglycine *t*-butyl ester (DAPT, HY-13027) was from MedChem Express.

### Flow cytometry

Cells were washed in phosphate buffered saline (PBS) and collected by centrifugation. The cells were stained with primary antibodies including CD44-FITC (560977, BD Pharmingen), CD24-Alexa 647 (56144, BD Pharmingen), c-Met-Alexa 647 (566014, BD Pharmingen), CD326-BB515 (565398, BD Pharmingen), CD133-PE (VII 70485, BD Pharmingen) at 4 °C for 30 min. After washing with PBS twice and re-suspended in 800 µL PBS, the cells were filtered through a 70 mm nylon mesh and carried out on a flow cytometer (BD Biosciences, Heidelberg, Germany). The viable and single cells were gated for analyses. BD CellQuest pro (BD Biosciences) and FlowJo (BD Bioscience) software were used to analyze the data.

### Transgenic mice, animal xenograft and orthotopic tumor model

Mice were housed at the animal core facility of the National Health Research Institutes (NHRI) and the University of Kaohsiung Medical University (UKMU), Taiwan. The facilities were approved by the National Association for Accreditation of Laboratory Animal Care, Taiwan, and is maintained in accordance with the regulations and standards of the NHRI and UKMU Animal Council’s procedural and ethical guidelines. Pdx-1-Cre, LSL-Kras^G12D^ and p53^Loxp/Loxp^ breeder mice, generated by Drs. Andrew M. Lowy, Tyler Jacks and Anton Berns [9], were obtained from the Mouse Models of Human Cancers Consortium (MMHCC) under material transfer agreements. KLF10^Loxp/Loxp^ (KLF10 L/L) mice were generously provided by Drs. John Hawse and Malayannan Subramaniam at the Mayo Clinic. All compound mutant mice were on a mixed genetic 129Sv × C57BL/6 background, and detailed genotyping protocols are available on request. Nonobese diabetic (NOD)/severe combined immunodeficient (SCID) mice (NOD. CB17-*Prkdc*^*scid*^/Jnarl) were purchased from the National Laboratory Animal Center, Taipei, Taiwan.

For the murine model of orthotopic pancreas tumor, a small left abdominal flank incision was made, and the spleen was exteriorized. A 1-mL syringe with a 29-gauge needle, in which Panc-1-Luc cells were suspended in PBS with VitroGel, (VHM01, Thewell, Bioscience) at a concentration of 1 × 10^6^ cells/50 μL, was inserted into the tail of pancreas. To prevent tumor cell leakage and bleeding, a cotton swab was held over the injection site for 1 min. The wound was sutured with 5-0 Chromic catgut (CC125, Shin N Med. Inst. Co). For in vivo limiting dilution analysis, tumor cells of 1 × 10^3^, 1 × 10^4^ and 4 × 10^4^ were mixed with VitroGel, (VHM01) at 1:1 ratio and injected subcutaneously into right flank of NOD/SCID mice. Tumor volume was measured twice every week with a caliper, and the volume was calculated using the formula of Tumor volume = 1/2 (length × width^2^) [10]. DAPT was prepared by dissolving in 30% dimethyl sulfoxide (DMSO) and diluted with PBS to 15 mg/kg/day and intraperitoneally injected 5 days every week for 2 weeks [11] before sacrifice. Metformin was dissolved in distilled water at dose of 120 mg/kg and intraperioneally injected for 3 weeks, 5 days a week. For evodiamine treatment, mice were given 15–30 mg/kg of evodiamine dissolved in DMSO, diluted in PBS, and intraperitoneally injected for 3 weeks, 5 days a week [12].

Mice were anesthetized by intraperitoneal administration of Attane (isoflurane, DP4900V22LA00), and tumor tissues were collected for further analysis. Pancreatic tissue samples were fixed in 10% buffered formalin overnight, washed with 1× PBS, and transferred to 70% ethanol before paraffin embedding, sectioning, and hematoxylin and eosin staining.

### Immunohistochemical (IHC)

Paraffin-embedded tissue sections were deparaffinized in xylene, rehydrated through a series of ethanol dilutions, and boiled for 15 min in 10 µM citrate buffer at pH 6.0. Endogenous peroxidase activity was suppressed using a peroxidase block (RE7148, Novolink) for 5 min. The tissue sections were then blocked using protein blocks (RE-7159, Novolink) and incubated overnight at 4 °C with antibodies against KLF10 (1:400, mouse anti-human monoclonal antibody, LTK BioLaboratories), Notch-3 (1:500, ab23426, Abcam, Cambridge, MA), Notch-4 (1:200, ab199295, Abcam). Antibody detection was performed using the Novolink Max Polymer Detection System (RE7280-K, Leica Biosystems).

### Immunoblotting

Cell extracts were prepared in lysis buffer (RIPA Cell Lysis Buffer 5×, RP05-100, Visual Protein, Taiwan) that contained a 1× protease inhibitor mixture (4693132001, Roche) and 1 mM phenylmethylsulfonyl fluoride. For western blot analysis, cell extracts were subjected to sodium dodecyl sulfate–polyacrylamide gel electrophoresis. The electro-transferred membrane (Protran ™ Nitrocellulose membrane NBA 085C001EA) was then incubated with the secondary antibody (92668072, IRDye® 680RD Donkey anti-Mouse IgG; 92632211 IRDye® 800CW Donkey anti-Rabbit IgG; LI-COR Biosciences) and was developed with a LI-COR Odyssey system (LI-COR). We used Notch-1 (1:1000, ab8952, Abcam), Notch-3 (1:1000, ab23426, Abcam), Notch-4 (1:1000, ab199295, Abcam), phospho-AMP-activated protein kinase (AMPK; 1:500, E-ab-21121, Elabscience), c-Myc (1:1000, E-ab-30975, Elabscience), Hes7 (1:1000, E-ab-18076, Elabscience), RBX1 (1:1000, E-ab-18881, Elabscience), FBXW7 (1:1000, E-ab-11064, Elabscience), DLL1 (1:1000, E-ab-66262, Elabscience), Hes1 (1:200, sc166410, Santa Cruz) and Hey L (1:200, sc81294, Santa Cruz) to detect Notch pathway associated protein markers. The KLF10 antibody was purchased from Abcam (1:1000, ab73537). β-Actin antibody (1:1000, E-ab-20094, Elabscience) at a 1:3000 dilution was used as control.

### Transfection and transduction

The construction of KLF10 expression vectors (HA-KLF10) has been described previously [13]. The stable downregulation of scramble and KLF10 plasmids (TRCN0000318921) in Panc-1 cells (Panc-1-pLKO-shKLF10) was achieved using a retrovirus-mediated RNA interference system (pSUPER.retro.puro, VEC-PRT-0001, OligoEngine) using shRNAs purchased from the National RNAi Core Facility of Academia Sinica (Taipei, Taiwan): 5′-GAACCCTCTCAAGTGTCAAAT-3′. Infected cell populations were selected using 2 mg/mL puromycin. The effect of shRNA knockdown was rescued by HA-KLF10 to rule out off-target effect [7]. As described previously [8], the Lenti-X™ Tet-One™ Inducible Expression Systems pLVX-TetOne-puro vector plasmids (631849, Clontech) was used for generation of stable doxycycline inducible clones of KLF10 insert under the TRE3G promoter in MiaPaCa cells (MiaPaCa-pLVX-KLF10). To determine whether Notch-3 or -4 are major downstream mediators of KLF10, stable KLF10 mRNA silencing clone was transiently transduced with *Notch-3* shRNA TRCN0000020236 (RNAiCORE, Sinica, Taiwan) and *Notch-4* shRNA TRCN0000020271 (RNAiCORE, Sinica) using TransIT-X2® Dynamic system (MIR 600, Mirus, Bio) for 48 h. The efficacy of transfection was confirmed through western blotting.

### Microarray assay and bioinformatics methods

The total RNA was extracted from Panc-1-pLKO and Panck-1-pLKO-shKLF10 cells by the RNeasy Mini Kit (74004, Qiagen, Hilden, Germany) according to the manufacturer’s instructions. RNA quality and integrity was measured by a 2100 Bioanalyzer (Agilent). Gene expression profiling was performed using the Illumina HumanHT-12 V4.0 chip (Illumina, Inc.) Microarray data normalization was performed using method of model based background correction (PMID: 20502629) in R. Differential gene expression levels between Panc-1-pLKO and Panc-1-pLKO-shKLF10 cell lines were identified by pairwise comparison analyses (≥ 1.5-fold threshold), and expression patterns were analyzed by hierarchical clustering. The canonical pathway analysis tool in Ingenuity Pathways Analysis (IPA, Ingenuity Systems) was used to identify cancer signaling pathways associated with differentially expressed genes. Gene set enrichment analysis (GSEA) was performed using the javaGSEA software developed at the Broad Institute (Cambridge, MA) and the MSigDB Hallmark gene set collection. Gene sets with a nominal of *p* value < 0.05 and false discovery rate (FDR) value ≤ 25% were considered to have significant enrichment. Microarray data are available in Gene Expression Omnibus (GEO) under accession number GSE218172 for gene expression.

### Chromatin immunoprecipitation (CHIP), ChIP-polymerase chain reaction (PCR) and quantitative real-time PCR

The EZ ChIP chromatin immunoprecipitation kit (#17-371, Sigma-Aldrich) was used according to the manufacturer’s protocol. Briefly, the cells were treated with 1% formaldehyde to cross-link proteins to DNA. The cells were lysed with protease inhibitors, sonicated to shear DNA into fragments and incubated with antibodies against KLF10, RNA polymerase III or anti-rabbit IgG overnight. The purified DNA and input genomic DNA were analyzed using real-time PCR. The primers used are as follow: promoter PCR sequences for *Notch-3*: Forward, TCACAGAGGAAGTGGGTTGC; Reverse, CAGCCTCAGACCTCAGACA. *Notch-4*: Forward, CCCCAAAGTTGTCCTGGGTT; Reverse, TCCTTGGGATGCAGGGAATG. The results were quantitated by Quantstudio 3 (Applied biosystems).

For RT-PCR, a reverse transcription kit (RR037Q, Takara Bio, San Jose, CA) and KAPA SYBR®FAST Mix (KK4600, KAPA Biosystems) were used as recommended by the manufacturer. The primers used were listed in Additional file 6: Table S1.

### Plasmid construction and promoter luciferase assay

The specific protein-1 (SP-1) binding sites over proximal promoter fragments, spanning within 1 Kb, of *Notch-3* and *-4* were cloned to drive the luciferase gene in the pGL4.1-based luciferase expression plasmid (#E6651, Promega; Additional file 3: Fig. S3A, B). Cells were transfected with individual reporter constructs and co-transfected with pRLTK (E2241, Promega), which constitutively expresses renilla luciferase, to normalize transfection efficiency. The ELF3/ELF3 S68A^mut^ expression vectors were constructed in the pcDNA 3.1(+) vector (V790-20, Addgene). After transfection for 24 h of HA-KLF10 and/or ELF3/ELF3 S68A^mut^ at various ratio, the promoter activities were determined using a dual-luciferase assay kit (E1910, Promega). Data are presented as the ratio of promoter reporter luciferase activity to control vector pGL4.1-enhancer luciferase activity.

Additional information on materials can be found in Additional file 7: Materials and methods.

## Results

### KLF10 deficiency correlated with accelerated pancreatic tumor growth

**Fig. 1:**
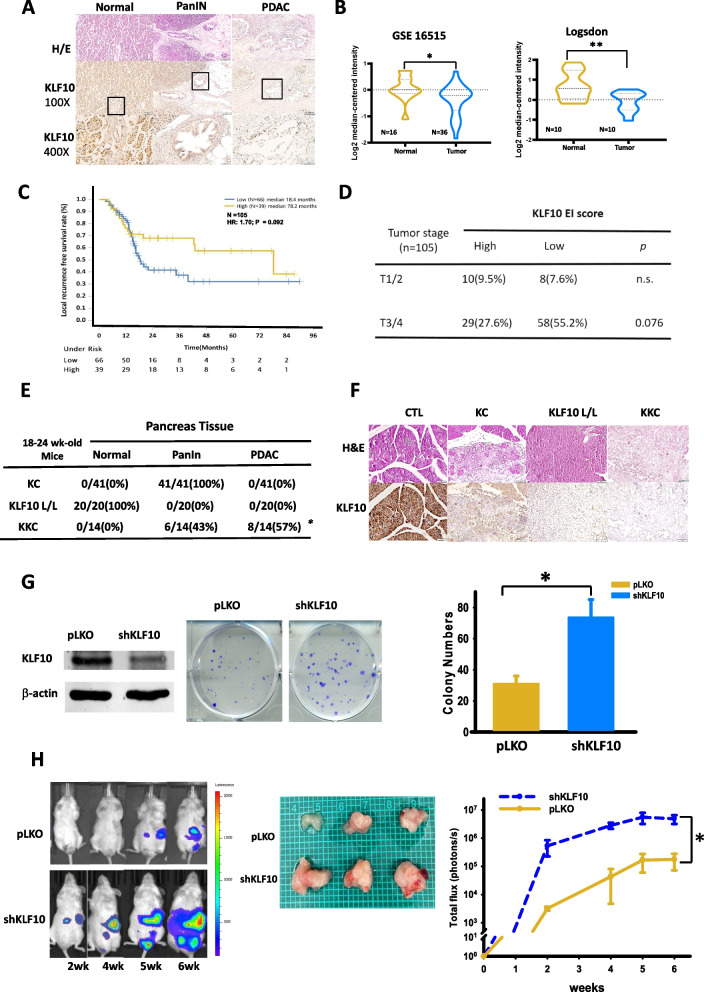
KLF10 deficiency correlated with accelerated pancreatic tumor growth. **A** Representative H&E (upper panel) and KLF10 immunostaining (middle and lower panel) of human pancreatic normal tissues (left panel), intraepithelial pancreatic neoplasm (PanIN, middle panel), and invasive pancreatic adenocarcinoma (PDAC, right panel). Original magnification: ×100 (mid-panel), ×400 (lower panel). **B** Violin plots of KLF10 transcript levels of pancreatic normal (yellow) versus tumor (blue) tissues from two representative databases of Gene Expression Omnibus 16515 (n = 52) and Oncomine Logsdon n = 20). *p < 0.05 and **p < 0.01, respectively. **C** Local recurrence-free survival curves of 105 patients of resected PDAC with low (n = 66) versus high (n = 39) expression of KLF10 immunostaining as described in “Materials and methods” (HR: 1.70, *p* = 0.092). **D** Tumor stage (T1/2 versus T3/4) of 105 patients of resected PDAC with high and low expression of KLF10 immunostaining as described in “Materials and methods”. For T3/4, *p* = 0.076. **E** Malignant progression to PanIN or PDAC of pancreas tissue in 18 to 24 week-old transgenic mice including Pdx-1-Cre/LSL-K-Ras^G12D^ (KC), Pdx-1-Cre/LSL-KLF10 (KLF10 L/L) and Pdx-1-Cre/LSL-Kras^G12D^/LSL-KLF10 (KKC) mice. *p < 0.05. **F** Representative H&E (upper panel) and KLF10 staining (lower panel) of pancreas tissue from KC, KLF10 L/L and KKC mice of 18 to 24 week-old. **G** Representative immunoblots (left panel) and colony formation (middle panel) of Panc-1 cells with vector control (pLKO) or KLF10mRNA silencing (shKLF10). Quantitative bar graphs (right panel) represent cumulated data from three independent experiments of colony formation assay. *p < 0.05. **H** Representative images of IVIS during 2–6 weeks after tumor implantation (left panel) and resected tumors at 6th week (middle panel) from orthotopic murine model of Panc-1 pLKO (yellow) and Panc-1-pLKO-shKLF10 (blue) as described in “Materials and methods”. Cumulated IVIS signal (right panel) of at least six mice in each experimental group that were injected with Panc-1-pLKO and Panc-1-pLKO-shKLF10 as indicated. Each point represents mean ± standard error (SE). *p < 0.05

Reduced KLF10 expression was noted in the tumor tissues of PDAC patients compared with normal pancreas tissue in immunohistochemistry (IHC) studies. Both cytosol and nuclei in normal pancreas and pancreatic intraepithelial neoplasm (PanIN) exhibited stronger staining than did tumor cells of PDAC (Fig. 1A). The findings are consistent with those of our previous study that involved a tumor/normal tissue paired-tissue array [3]. Gene expression profiles of PDAC also showed reduced KLF10 transcripts of tumor tissue compared with normal pancreas tissue in several databases (Fig. 1B). A trend of faster local recurrence was evident in 105 patients with resected PDAC (Fig. 1C; 18.4 vs 78.2 months, *p* = 0.092) [ClinicalTrials.gov. identifier: NCT 00994721]. A larger tumor size (Fig. 1D, *p* = 0.076) was found in patients with low expression of KLF10 [14]. Despite a non-significant correlation with local tumor control and tumor size, partly due to small sample size, KLF10 was a significant prognostic factor for overall survival (*p* = 0.013) of this cohort of patients with resected PDAC [14]. These results suggest that KLF10 may modulate tumor growth of PDAC.

In the murine model of spontaneous pancreatic cancer, we found that all LSL: Kras^G12D^; Pdx1-Cre (KC) mice developed PanIN lesions without invasive carcinoma when they were 18–24 week-old (Fig. 1E). KLF10-knockout (KLF10 L/L) mice of similar age did not have pancreatic malignancy. However, invasive PDAC developed in over 50% of 18–24 week-old KC mice with additional KLF10 depletion (KKC mice) at the frontiers of PanIN lesions (Fig. 1E). In addition, IHC revealed prominent ductal dysplasia, tumor invasion, and fibrotic stroma with down-regulated KLF10 expression in the majority of the pancreatic tumors of KKC mice (Fig. 1F). KLF10 L/L mice, by contrast, exhibited no malignant transformation, and KC mice had various levels of PanIN lesions with moderate KLF10 immunostaining (Fig. 1F).

An in vitro clonogenic assay using Panc-1 cells with KLF10 mRNA knockdown (Panc-1-pLKO-shKLF10) revealed an increased colony number and size when compared with Panc-1 cells of the vector control (Panc-1-pLKO; Fig. 1G). Similar findings were observed in murine cell lines established from KPC (*Pdx1-Cre*; *Kras*^*G12D*^; *Trp53*^*LoxP/LoxP*^) mice versus those from KKPC (*Pdx1-Cre*; *Kras*^*G12D*^; *Trp53*^*LoxP/LoxP*^; *KLF10*^*L/L*^) mice (Additional file 1: Fig. S1A). We used a murine orthotopic tumor model by implanting Panc-1-Luc cells in the pancreas, as described in “Materials and methods”. The tumor growth rate was significantly higher in mice that received implantation of Panc-1-pLKO-shKLF10 compared with those that received Panc-1-pLKO (Fig. 1H). These findings suggest that KLF10 plays a pivotal role in pancreatic tumorigenesis.

### KLF10 modulated stem cell phenotypes of PDAC

**Fig. 2:**
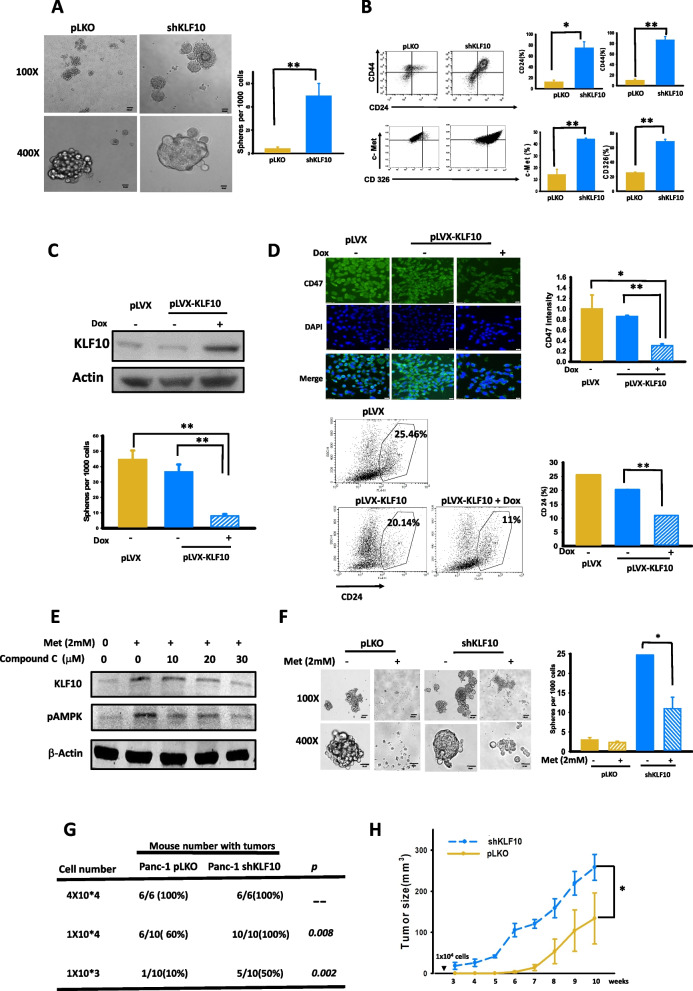
KLF10 modulated stem cell phenotypes of PDAC. **A** Representative pictures of sphere formation (left panel) of Panc-1-pLKO and Pan-1-pLKO-shKLF10 cells. Original magnification: (left upper panels) 1 × 100, (left lower panels) 1 × 400. Quantitative bar plots (right panel) represent mean ± SE of cumulated data from three independent experiments. **p < 0.01. **B** Representative flow cytometry (left panel) of stem cell markers including CD44, CD24, c-MET and CD326 of Panc-1-pLKO and Panc-1-pLKO-shKLF10. Quantitative bar plots (right panel) represent mean ± SE of cumulated data from three independent experiments of flow cytometry measuring each stem cell markers. *p < 0.05 and **p < 0.01, respectively. **C** (Upper panel) immunoblots of KLF10 expression in MiaPaCa cells with conditional overexpression of KLF10 (pLVX-KLF10) as described in “Materials and methods”. Dox represents doxycylin. β-Actin was used as internal control. (Lower panel) Quantitative bar plots of sphere formation from three independent data of MiaPaCa cells with pLVX vector control or MiaPaCa-pLVX-KLF10 treated without or with Dox. **p < 0.01. **D** (Left upper panels) immunofluorescence staining of CD47 (green) in MiaPaCa-pLVX or MiaPaCa-pLVX-KLF10 without or with Dox. Nuclei were counterstained with DAPI. Quantitative bar plots (right upper panel) represent mean ± SE from three independent experiments. *p < 0.05 and **p < 0.01, respectively. (Lower left panels) representative flow cytometry of CD24 expression of MiaPaCa-pLVX, and MiaPaCa-pLVX-KLF10 without or with Dox treatment. (Lower right panel) quantitative bar plots represent mean ± SE from three independent experiments. **p < 0.01. **E** Representative immunoblots of KLF10 and phospho-AMPK in Panc-1 cells treated without or with 2 mM metformin and 0–30 µM Compound C as described in “Materials and methods”. **F** Representative images, ×100 (left upper panels) and ×400 (left lower panel), of sphere formation from Panc-1-pLKO and Panc-1-pLKO-shKLF10 treated without or with 2 mM metformin as described in “Materials and methods”. Quantitative bar plots (right panel) of mean ± SE from cumulated data of three independent experiments. *p < 0.05. **G** In vivo limiting dilution assay. Number of mice with tumor growth after implanted subcutaneously with Panc-1-pLKO and Panc-1-LKO-shKLF10 cells of various cell number indicated as described in “Materials and methods”. **H** Tumor growth curves of mice after 1 × 10^4^ cells of Panc-1-pLKO and Panc-1-pLKO-shKLF10 implanted. Each point represents mean ± SE of cumulated data from at least six mice. *p < 0.05

We evaluated whether KLF10 expression modulates stem cell phenotypes of PDAC. Sphere formation ability was measured and compared between Panc-1 cells with and without KLF10 mRNA depletion. The number of spheres formed was significantly higher for Panc-1-pLKO-shKLF10 cells compared with Panc-1-pLKO cells when using the criteria described in “Materials and methods” (Fig. 2A). Induction of KLF10 overexpression significantly suppressed the activity of sphere formation in Panc-1 cells (Fig. 2C and Additional file 1: Fig. S1C).

We measured the co-expression of CD24, CD44, c-Met, and CD326 to detect pancreatic cancer stem cells. Panc-1-pLKO-shKLF10 cells had a three- to five fold increase in stem cell marker expression compared with Panc-1-pLKO cells (Fig. 2B). Similar observations were made for murine cell lines derived from KPC versus those from KKPC mice (Additional file 1: Fig. S1B). In addition, conditionally overexpressing KLF10 in MiaPaCa cells revealed that the levels of CD47 [15], CD24, and CD44 expression on tumor cells were reduced by more than 50% (Fig. 2D and Additional file 1: Fig. S1D).

Our previous study revealed that metformin, an oral anti-diabetic agent, increased the radiation sensitivity of PDAC cells by elevating KLF10 expression [8]. AMP-activated protein kinase (AMPK) was demonstrated to activate and stabilize KLF10 via phosphorylation at Thr189 [16]. In this study, we found that KLF10 expression was modulated by metformin via phosphorylating AMPK. Compound C, an AMPK inhibitor, suppressed AMPK phosphorylation and KLF10 expression (Fig. 2E). Sphere formation in Panc-1-pLKO-shKLF10 cells could be reduced by metformin, which elevated KLF10 expression (Fig. 2F).

We used in vivo limiting dilution assay to evaluate tumorigenic ability. When a 1 × 10^4^ or lower cell amount was implanted, the number of mice with tumor formation differed significantly between Panc-1-pLKO and Panc-1-pLKO-shKLF10 cells (Fig. 2G). Tumor size was significantly larger in the mice implanted with 1 × 10^4^ of Panc-1-pLKO-shKLF10 cells than in those implanted with Panc-1-pLKO cells (Fig. 2H).

These results suggest that KLF10 is significantly associated with stem cell phenotypes in PDAC tumorigenesis and progression, which might be reversed by metformin.

### KLF10 deficiency facilitated the Notch signaling pathways

**Fig. 3:**
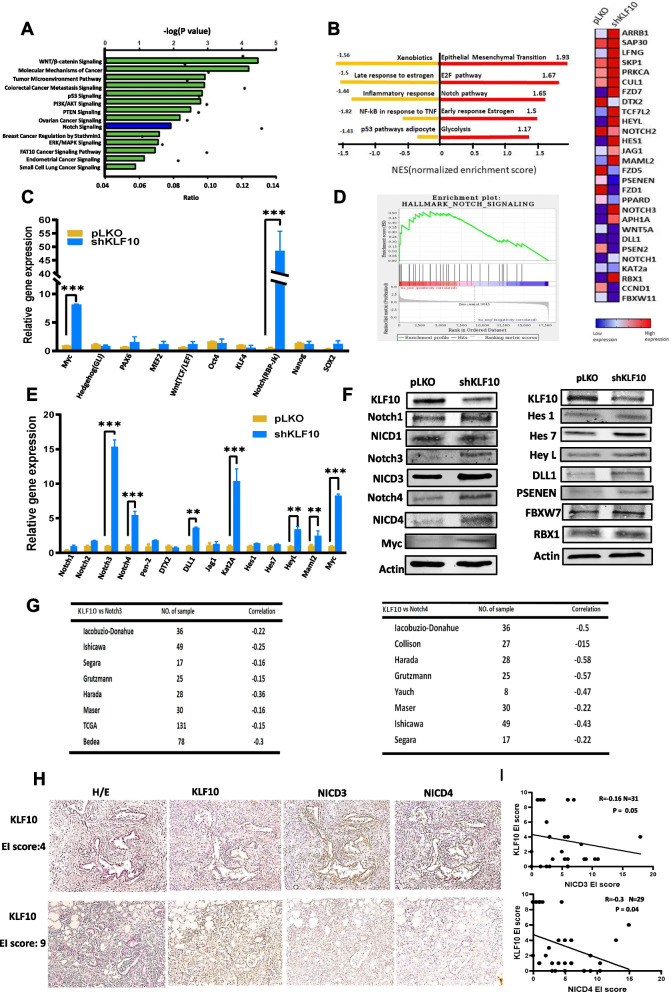
KLF10 deficiency facilitated the Notch signal pathway. **A** The most differentially expressed “cancer signaling pathways” associated with KLF10 depletion in Panc-1 cells by IPA. Graphs in green or blue show category scores as − log [p-value]. Ratio (black dot) indicates the molecules from the data set that map to the pathway listed divided by the total number of molecules that map to the pathway from within the IPA database. **B** The most enriched pathways associated with Panc-1-pLKO-shKLF10 cells by GSEA analysis. Red indicates positive and yellow indicates negative normalized enrichment score. **C** Quantitative RT-PCR of candidate signal pathways molecules related to stem cell phenotypes were measured in Panc-1-pLKO (yellow) and Panc-1-pLKO-shKLF10 (blue) cells. Data were presented in mean ± SE from cumulated results of three independent experiments. ***p < 0.005. **D** GSEA analysis of Panc-1-pLKO-shKLF10 versus Panc-1-pLKO showing upregulated HALLMARK_NOTCH_SIGNALING (left panel). Heat map of Notch signal pathway molecules expression of Panc-1-pLKO and Panc-1-pLKO-shKLF10 cells in GSEA analysis (right panel). **E** Quantitative RT-PCR of candidate molecules of Notch signal pathway in Panc-1-pLKO (yellow) and Panc-1-pLKO-shKLF10 (blue) cells. Data were presented in mean ± SE from cumulated results of three independent experiments. **p < 0.01 and ***0.005, respectively. **F** Representative immunoblots of Notch signal pathway molecules in Panc-1-pLKO and Panc-1-pLKO-shKLF10 cells. β-Actin was used as internal control. **G** Representative databases from Oncomine showing mild to moderate inverse correlation between the transcript levels of KLF10 versus Notch-3 (left panel); and KLF10 versus Notch-4 (right panel). **H** (Left panel) representative IHC staining of KLF10, NICD-3 and -4 in tumor tissues from two patients of PDAC showing low (upper panel, EI score = 4) and high (lower panel, EI score = 9) KLF10 expression. (Right panel) correlation of KLF10 versus NICD-3 (upper panel, n = 31) and KLF10 versus NICD-4 (lower panel, n = 29) expression on IHC in pancreatic tumors tissues from the cohort mentioned in “Materials and methods”. The correlation coefficients R were − 0.16 and − 0.3 with *p* = 0.05 and 0.04, respectively

A microarray analysis was used to identify differentially expressed genes in Panc-1 with or without KLF10 depletion as described in “Materials and methods”. After KLF10 depletion, an ingenuity pathway analysis (IPA) revealed that pathways, including the Wnt and Notch signaling pathways, were highly upregulated in the “cancer signal pathway” profile (Fig. 3A). A gene set enrichment analysis (GSEA) revealed that the Notch signaling pathway, which may modulate stem cell phenotypes, was one of the most differentially upregulated by Panc-1-pLKO-shKLF10 cells (Fig. 3B, D). We measured representative transcripts of candidate molecules from 10 signaling pathways essential to cancer stem cell survival to understand the signals governing stem cell homeostasis in Panc-1 cells with or without KLF10 deficiency (Fig. 3C and Additional file 6: Table S1A). Notch signaling was one of the most upregulated in Panc-1-pLKO-shKLF10 compared with Panc-1-pLKO cells. Since Notch signal transcript was the most upregulated one in the 10-candidate signaling pathway of cancer stem cell and in both IPA and GSEA studies, we chose Notch pathway rather than WNT/b-catenin or EMT pathway for further investigation.

The Notch signaling molecules were validated in RNA and protein expression levels in Panc-1 cells with or without KLF10 depletion (Fig. 3E, F, Additional file 2: Fig. S2A and Additional file 6: Table S1B) as well as in MiaPaCa cells with or without ectopic KLF10 overexpression (Additional file 2: Fig. S2B, C). In Panc-1-pLKO-shKLF10 cells, we found increased expression of the Notch ligands, receptors, activated intracellular domain, activating enzymes and down-stream effectors, which correlates with the heatmap of the Notch signaling pathway from GSEA (Fig. 3D, right panel). Among all, Notch-3 and Notch-4 in both full length and activated forms (i.e., Notch-3 and -4 intracellular domains, NICD-3/4) were all significantly upregulated in Panc-1-pLKO-shKLF10 compared with Panc-1-pLKO cells (Fig. 3E, 3F and Additional file 2: Fig. S2A).

Transcriptomic data from Oncomine revealed a low-to-moderate inverse correlation between expression of KLF10 and Notch-3/4 in several databases (Fig. 3G). IHC of 31 and 29 tumor specimens, respectively, from the cohort of PDAC patients described in “Materials and methods”, also showed a trend of inverse correlation between KLF10 and NICD-3/4 (Fig. 3H).

We concluded that the Notch signaling pathway is activated by KLF10 deficiency in PDAC.

### KLF10 suppressed *Notch-3* and *Notch-4* gene transcription

**Fig. 4:**
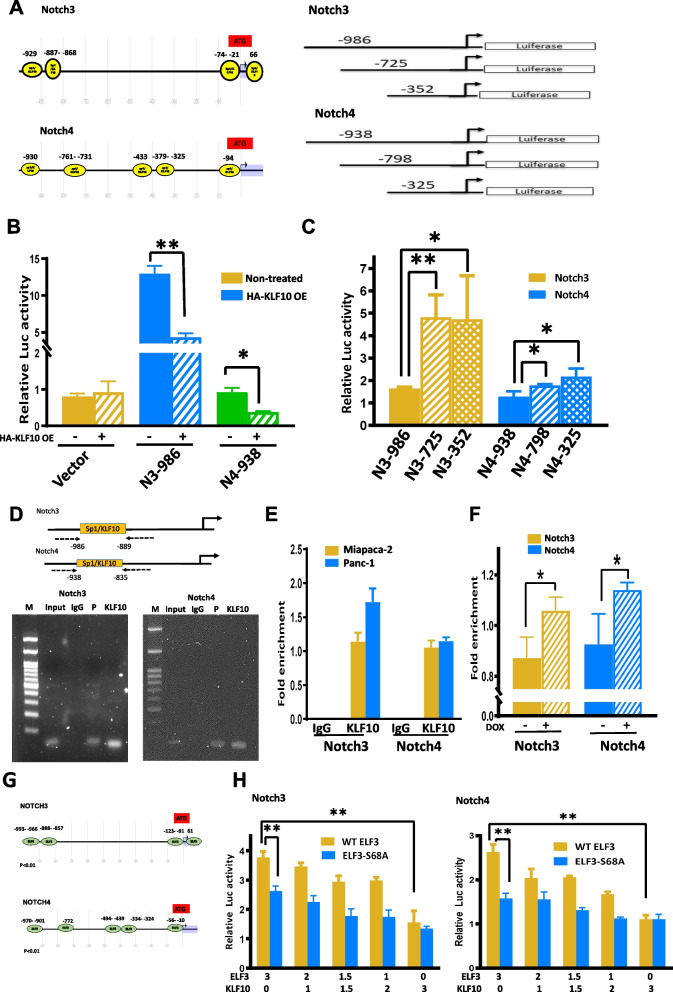
KLF10 competed with ELF3 and transcriptionally suppressed *Notch-3 and -4*. **A** Primer maps with predicted SP-1/KLF10 binding sites (left panel, predicted *p* < 0.001) and various designed deletion (right panel) of promoter regions of *Notch-3* and *-4* for luciferase reporter assay. **B** Luciferase activity of Panc-1 cells transfected with vector control (yellow), *Notch-3(blue), and Notch-4(green)* gene promoter plasmids without (filled) or with (diagonal stripe) hemagglutinin (HA) tagged-KLF10 transfection (OE). The bar graphs were mean ± SE from cumulated data of three independent experiments. *p < 0.05 and **p < 0.01, respectively. **C** Luciferase activity of Panc-1 cells, transfected with *Notch-3* (yellow) or *Notch-4* (blue) gene promoter plasmids with full length (filled) or with various deletion (diagonal stripe, diagonal check) indicated. The bar graphs were mean ± SE from cumulated data of three independent experiments. *p < 0.05 and **p < 0.01, respectively. **D** (Upper panel) primer maps of *Notch-3/4* gene promoters for CHIP-PCR assay. (Lower panel) CHIP assay. DNA fragments of Panc-1 cells were immune-precipitated with KLF10, IgG or positive control (P) followed by PCR amplification of the *Notch-3/4* gene promoter region that contains KLF10 binding sites (input), as described in “Materials and methods” section. **E** Quantitative PCR of the *Notch-3/4* gene promoter region using samples from MiaPaCa (yellow) or Panc-1 (blue) cells prepared as described in **D**. The bar graphs were mean ± SE from cumulated data of three independent experiments. **F** MiaPaCa cells with conditional over-expression of KLF10 under Dox treatment were immuno-precipitated with KLF10 antibody and quantitative PCR of the *Notch-3* (yellow) and *Notch-4* (blue) gene promoter regions. The bar graphs were mean ± SE from cumulated data of three independent experiments. *p < 0.05. **G** Primer maps of predicted ELF3 binding sites on the promoter regions of *Notch-3* (upper panel) and *Notch-4* (left panel) genes. **H** Luciferase reporter assays of *Notch-3* (left panel) and *Notch-4* (right panel) under conditions of various combination of HA-KLF10 and ELF3 (yellow) and ELF3-mutant (ELF3-S68A, blue) as indicated. The bar graphs were mean ± SE from cumulated data of three independent experiments. **p < 0.01

To delineate the molecular mechanisms of KLF10 in regulating *Notch-3* and *Notch-4* transcription, we evaluated candidate binding sites in the promoter region of the human *Notch-3* and *Notch-4* genes by using the Eukaryotic Promoter Database (Swiss Institute of Bioinformatics, Lausanne, Switzerland). Since KLF10 belongs to the specificity protein-1 (SP-1)-like transcription factor family, over 20 putative SP-1 binding elements were recognized from the gene promoters of human *Notch-3* and *Notch-4* with predicted p < 0.01 (Fig. 4A, left panel, predicted p < 0.001). To validate if KLF10 indeed acted through these elements, the luciferase reporter genes were constructed to be driven by the human *Notch-3/4* gene promoter region containing the wild-type and deleted SP-1 binding motifs, respectively (Fig. 4A, right panel; Additional file 3: Fig. S3B, C). Luciferase activities of wild-type *Notch-3/4* gene promoters were significantly decreased by HA-KLF10 in Panc-1 cells (Fig. 4B). However, reduced luciferase activity of *Notch-3/4* gene promoters with deletion of − 986 to − 726 and − 938 to − 797 was not observed (Fig. 4C).

To demonstrate that KLF10 directly bound to *Notch-3* and *Notch-4* gene promoters, we conducted the Chromatin Immunoprecipitation (ChIP) assay in Panc-1 cells with an anti-KLF10 antibody and polymerase chain reaction (PCR) primers spanning each SP-1-binding site of the *Notch-3/4* gene promoter regions (Fig. 4D, upper panel; Additional file 3: Fig. S3A). We observed physical binding of KLF10 with the promoters of *Notch-3* and *Notch-4* genes (Fig. 4D, lower panel). CHIP-PCR demonstrated physical binding of KLF10 with promoters of *Notch-3* and *-4* in Panc-1 and MiaPaCa cells (Fig. 4E). Overexpressing KLF10 in MiaPaCa cells exhibited enhanced binding of KLF10 with promoters of *Notch-3* and *Notch-4* genes (Fig. 4F).

Because E74-like ETS transcription factor 3 (ELF3) was reported to bind to *Notch-3* gene promoter with transcription regulation in K-RAS-mediated lung adenocarcinoma [17], we evaluated the interaction of KLF10 and ELF3 in Panc-1 cells. The predicted ELF3-binding sites of *Notch-3/4* gene promoters highly overlapped with those of KLF10 (Fig. 4G). We constructed plasmids expressing wild-type and mutated ELF3, as described in “Materials and methods” (Additional file 3: Fig. S3D). Luciferase expression in the *Notch-3/4* gene promoter-driven reporter assay increased in proportion with increasing amount of ELF3 over KLF10. The phenomenon was not as significant in competition assay using mutated ELF3 with KLF10 (Fig. 4H). Phosphorylation of serine 68 of ELF3 was demonstrated to participate in nuclear import, accumulation and retention of ELF3 in Kras mutated lung adenocarcinoma [17] The partial competition between S68A ELF3 mutant and KLF10, especially in *Notch-3* gene promoter activity, may be attributed to multiple binding sites of ELF3 on *Notch-3* promoter with various affinity and inherent levels of KLF10/ELF3 in Panc-1 cells. Several GEO databases of pancreatic adenocarcinoma revealed a positive correlation of *ELF3* with *Notch-3* and *-4* transcripts (Additional file 3: Fig. S3E, left panel). An inverse correlation of *ELF3* and *KLF10* expression was also found (Additional file 3: Fig. S3E, right panel).

The findings suggest that *ELF3* and *KLF10* competed for activation and suppression, respectively, of the *Notch-3* and *-4* promoter activity (Fig. 4H).

### Inhibition of Notch signal reversed stem cell phenotypes induced by KLF10 deficiency

**Fig. 5:**
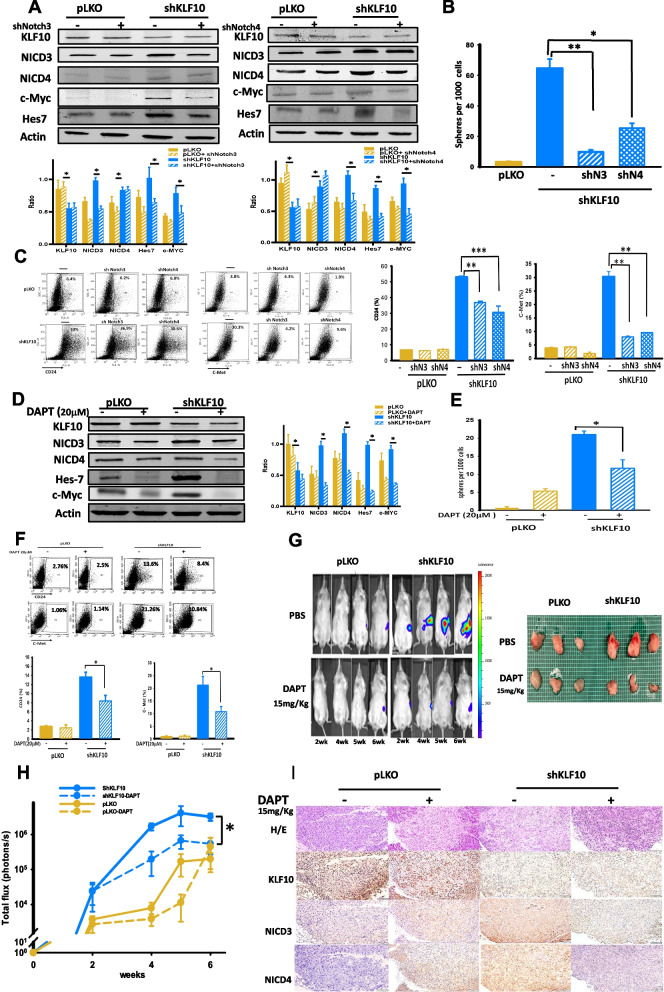
Inhibition of Notch signal reversed stem cell phenotypes induced by KLF10 depletion. **A** (Upper panels) representative immunoblots of KLF10 and Notch signal pathway molecules as indicated in Panc-1-pLKO or Panc-1-pLKO-shKLF10 cells without or with *Notch-3* (left panel) or *Notch-4* (right panel) genes silencing. β-Actin was used as internal control. (Lower panels) the bar graphs were mean ± SE from cumulated data of three independent experiments. Levels of signal molecule expression in Panc-1-pLKO (yellow) or Panc-1-pLKO-shKLF10 (blue) cells without (filled) or with Notch 3/4 (shN3/4, diagonal stripe) receptor mRNA silencing were measured. *p < 0.05. **B** Sphere formation of Panc-1-pLKO (yellow) or Panc-1-pLKO-shKLF10 (blue) cells without (filled) or with Notch 3 (shN3, diagonal stripe) or Notch 4 (shN4, diagonal check) receptor mRNA silencing. The bar graphs were mean ± SE from cumulated data of three independent experiments. *p < 0.05 and **p < 0.01, respectively. **C** (Left panels) representative flow cytometry of CD24 and c-Met on Panc-1-pLKO (upper panels) and Panc-1-pLKO-shKLF10 (lower panels) cells without or with *Notch-3/4* mRNA silencing as indicated. (right panels) Expression of CD24 (left panel) and c-Met (right panel) on Panc-1-pLKO (yellow) or Panc-1-pLKO-shKLF10 (blue) cells without (filled) or with *Notch-3* (sN3, diagonal stripe) or *Notch-4* (sN4, diagonal check) mRNA silencing. The bar graphs were mean ± SE from cumulated data of three independent experiments. **p < 0.01 and ***p < 0.005, respectively. **D** (Left panel) representative immunoblots of KLF10 and Notch signal molecules in Panc-1-pLKO or Panc-1-pLKO-shKLF10 cells without or with 20 µM DAPT treatment. β-Actin was used as internal control. (Right panel) the bar graphs were mean ± SE from cumulated data of three independent experiments. Levels of signal molecule expression in Panc-1-pLKO (yellow) or Panc-1-pLKO-shKLF10 (blue) cells without (filled) or with DAPT (diagonal stripe) treatment were measured. *p < 0.05. **E** Sphere formation of Panc-1-pLKO (yellow) or Panc-1-pLKO- shKLF10 (blue) cells without (filled) or with (diagonal stripe) 20 µM DAPT treatment. The bar graphs were mean ± SE from cumulated data of three independent experiments. *p < 0.05. **F** (Upper panels) representative flow cytometry of CD24 and c-Met on Panc-1-pLKO (left panels) and Panc-1-pLKO-shKLF10 (right panels) cells without or with DAPT treatment as indicated. (Lower panels) expression of CD24 (left panel) and c-Met (right panel) on Panc-1-pLKO (yellow) or Panc-1-pLKO-shKLF10 (blue) cells without (filled) or with (diagonal stripe) 20 µM DAPT treatment. The bar graphs were mean ± SE from cumulated data of three independent experiments. *p < 0.05. **G** Representative IVIS images of mice at 2–6 weeks after implanting orthotopically with Panc-1-pLKO or Panc-1-pLKO-shKLF10 cells without or with DAPT 15 mg/kg intra-peritoneally for 3 weeks as described in “Materials and methods” (left panel). Representative photos of tumors resected from mice experiments mentioned above (right panel). **H** Cumulated IVIS signal of at least six mice in each experimental group that were implanted with Panc-1-pLKO (yellow) and Panc-1-pLKO-shKLF10 (blue) without (solid) or with (dashed line) DAPT treatment for 3 weeks as indicated. Each point represents mean ± SE. *p < 0.05. **I** Representative H&E (upper panel) and IHC staining, as described in “Materials and methods”, of KLF10, NICD-3 and -4, as indicated, in tumors resected from mice experiments of **G**

To demonstrate that the Notch signaling pathway contributed to KLF10 in modulating stem cell phenotypes of PDAC, we silenced mRNA of *Notch-3* or* -4* genetically in Panc-1-pLKO-shKLF10 cells. The immunoblots revealed that Notch-3, Notch-4, NICD-3, NICD-4, Hes 7, and c-Myc were elevated by KLF10 depletion in Panc-1 cells. The phenomenon was reversed by genetically depleting Notch-3 and -4, respectively (Fig. 5A). Sphere formation and expression levels of CD24 and c-Met in Panc-1-pLKO-shKLF10 were also reversed by *Notch-3/4* knockdown (Fig. 5B, C). Despite small difference in elevating Notch-3 and -4 levels by KLF10 knock-down, there was no evident disparity of biologic outcomes of Notch-3/4 depletion in Panc-1 shKLF10 cells. Pharmacologically, *N*-[*N*-(3,5-difluorophenacetyl)-l-alanyl]-*S*-phenylglycine *t*-butyl ester (DAPT, a γ-secretase inhibitor) was used to suppress Notch signal transduction. NICD-3 and -4 as well as Hes 7, and c-Myc were down-regulated in Panc-1-pLKO-shKLF10 after DAPT treatment (Fig. 5D). Since the level of presenilin enhancer, gamma-secretase subunit (PSENEN, PEN-2), was elevated after KLF10 mRNA silencing (Fig. 3F), the therapeutic effect of DAPT was more prominent on Panc-1-pLKO-shKLF10 compared with that on Pan-1pLKO cells (Fig. 5D). The enhanced sphere formation and stem cell markers of CD24 and c-Met in Panc-1-pLKO-shKLF10 were suppressed by DAPT (Fig. 5E, F, Additional file 4: Fig. S4A, B).

In the orthotopic murine model, the tumor growth of Panc-1-pLKO-shKLF10 cells was ameliorated by DAPT treatment (Fig. 5G, H). The histology of tumors from mice implanted with Panc-1-pLKO-shKLF10 after DAPT treatment revealed significant loss of immune-labeling of NICD-3 and -4 but no effect on KLF10 expression (Fig. 5I).

We concluded that the suppression of the Notch signaling pathway genetically or pharmacologically might reverse the stem cell phenotypes of PDAC with KLF10 deficiency.

### Metformin and evodiamine cooperatively reduced stem cell phenotypes and tumor growth of PDAC

**Fig. 6:**
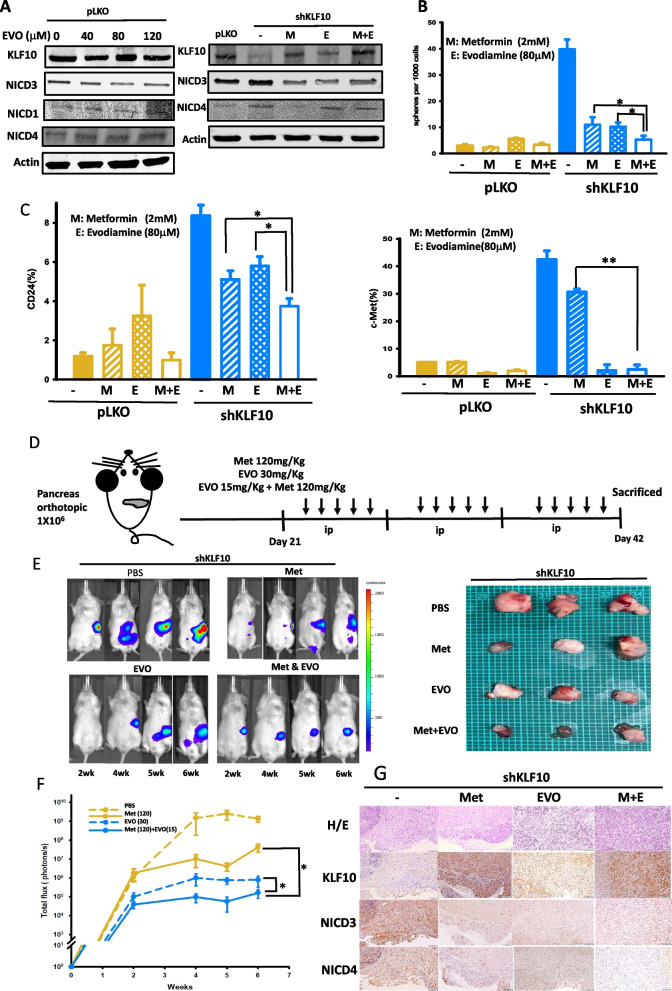
Metformin and evodiamine cooperatively reduced stem cell phenotypes and tumor growth of PDAC. **A** Representative immunoblots of KLF10 and NICD-1, -3, and -4 in Panc-1 cells treated with various dosage of evodiamine (EVO) as indicated. β-Actin was used as internal control (left panel). Representative immunoblots of KLF10 and NICD-3 and -4 in Panc-1-shKLF10 treated with combination of 2 mM metformin (M) and/or 80 µM evodiamine (E) (right panel). **B** Sphere formation of Panc-1-pLKO (yellow) and Panc-1-pLKO-shKLF10 (blue) without treatment (filled) or treated with metformin alone (diagonal stripe) evodiamine alone (diagonal check) or with combination treatment (unfilled). The bar graphs were mean ± SE from cumulated data of three independent experiments. *p < 0.05. **C** Expression of CD24 (left panel) and c-MET (right panel) on Panc-1-pLKO (yellow) and Panc-1-pLKO-shKLF10 (blue) without treatment (filled), treated with metformin alone (diagonal stripe), with evodiamine alone (diagonal check), or with combination treatment (unfilled). The bar graphs were mean ± SE from cumulated data of three independent experiments. *p < 0.05 and **p < 0.001 respectively. **D** Schema of orthotopic murine model treated with metformin (120 mg/kg) and/or evodiamine (15 or 30 mg/kg). **E** (Left panel) representative IVIS images of mice bearing Panc-1-shKLF10 and treated with metformin (Met) and/or evodiamine (EVO). (Right panel) representative photos of tumors resected from murine experiments mentioned above. **F** Cumulated IVIS signal of at least six mice in each experimental group that were implanted with Panc-1-shKLF10 and received PBS (dashed yellow), metformin alone (solid yellow), evodiamine alone (dashed blue), or combination (solid blue) for 3 weeks, as indicated. Each point represents mean ± SE. *p < 0.05 and **p < 0.01. **G** Representative H&E (upper panel) and IHC staining, as described in “Materials and methods”, of KLF10, NICD-3 and NICD-4, as indicated, in tumors resected from mice experiments of **E**

Because of the notorious toxicity and lack of survival benefit of γ-secretase inhibitors in clinical application [18, 19], we evaluated evodiamine, a specific Notch-3 inhibitor that was reported to reverse tumor growth in non-small cell lung cancer with minimal side effects [12]. In Panc-1 cells, evodiamine exhibited relative suppression of NICD-3 but not of NICD-1 and -4 (Fig. 6A, left panel). Because of the low-to-moderate inverse correlation between KLF10 and Notch-3, we used metformin and evodiamine combination treatment in Panc-1 cells to increase KLF10 while suppressing Notch-3 signaling for better therapeutic outcomes. The combination of metformin and evodiamine in Panc-1-pLKO-shKLF10 suppressed NICD-3 and -4 and elevated KLF10 expression (Fig. 6A, right panel). The sphere-forming activity and the stem cell markers expression decreased by concomitant metformin and evodiamine compared to either reagent alone (Fig. 6B, C). The murine orthotopic model treated with the combination of metformin and evodiamine showed reduced tumor formation of Panc-1-pLKO-shKLF10 without evident body weight loss or toxicity (Fig. 6D–F and Additional file 5: Fig. S5). IHC of tumors in mice implanted orthotopically with Panc-1-pLKO-shKLF10 and treated with the combination of metformin and evodiamine revealed increased KLF10 and reduced NICD-3 and -4 expression (Fig. 6G).

**Fig. 7:**
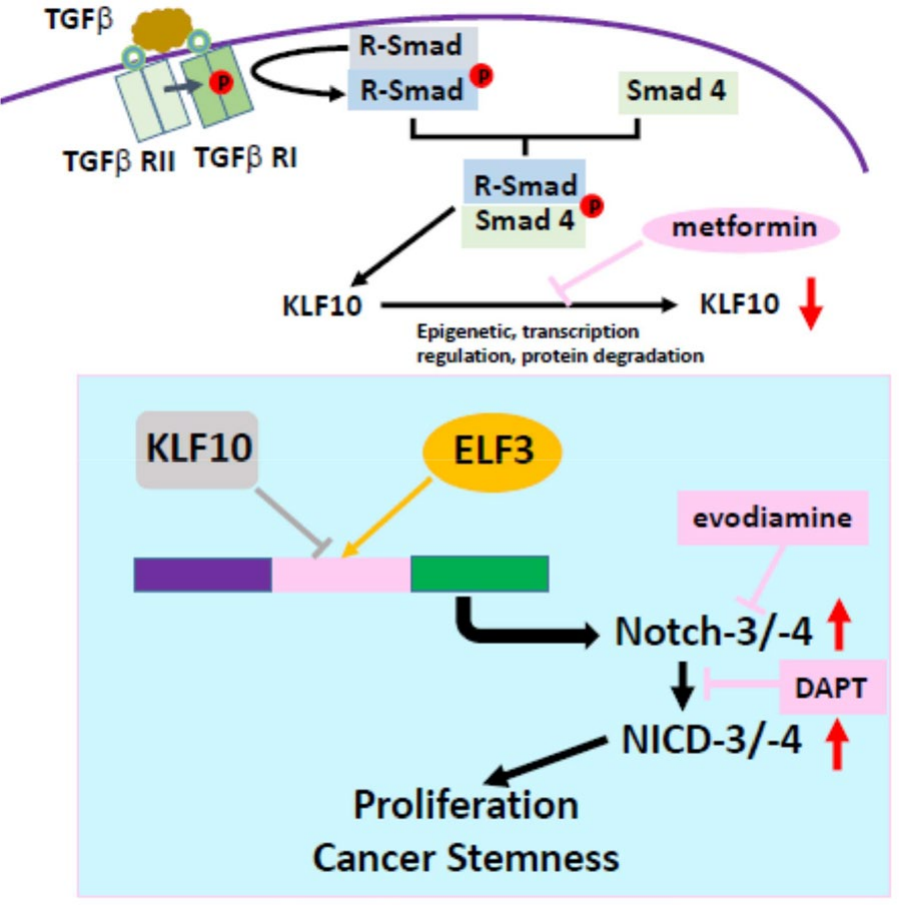
KLF10/Notch signal pathway in modulating proliferation and stemness of PDAC. Loss of KLF10, due to epigenetic, transcriptional regulation or protein degradation in PDAC, interferes the balance of ELF3 and KLF10 competing for activation and suppression, respectively, of the *Notch-3* and *-4* promoter activity which modulates cancer cell proliferation and stemness phenotypes. Elevating KLF10 expression level by metformin, upregulating Notch 3 transcription by evodiamine or NICD by DAPT, ameliorates PDAC progression induced by KLF10 deficiency

The results suggest that the combination of metformin and evodiamine suppressed tumor growth of KLF10-deficient PDAC cells by elevating KLF10 and suppressing Notch-3/4 signals without any substantial toxicity (Fig. 7).

## Discussion

PDAC is one of the most complex and aggressive malignancies, exhibiting rapid progression, and is resistant to most cancer therapies. A recent study revealed that cancer cell stemness in PDAC is a major factor that contributes to tumorigenesis, progression, and therapy resistance and are associated with poor clinical outcomes [20]. Our previous studies have shown that KLF10 is not only a prognostic indicator for survival but also a predictor for therapeutic outcomes in PDAC [3, 7, 8]. This study demonstrated that KLF10 regulated sphere formation, expression of stem cell markers and tumorigenesis in PDAC cells. The IPA and GSEA of the microarray study revealed that the Notch signaling pathway was significantly upregulated in Panc-1-pLKO-shKLF10. KLF10 bound to the promoter regions of *Notch-3* and *-4* genes*,* and transcriptionally suppressed Notch signal transduction. Genetically depleting Notch-3/4, or pharmacologically suppressing Notch signal by γ-secretase inhibitor ameliorated the stem cell phenotypes and tumor growth induced by KLF10 deficiency in PDAC cells. The combination of metformin, which upregulated KLF10 expression, and evodiamine, a non-toxic Notch-3 inhibitor, did not only reduced sphere formation activity and stem cell markers expression in Panc-1-pLKO-shKLF10 cells, but also exhibited its therapeutic efficacy in suppressing the tumor growth of the Panc-1shKLF10 cells in orthotopic implant mice.

Unlike many other signaling pathways that can be transduced via kinase cascades, the Notch signaling pathway lacks a mediator to amplify signal. Several studies have found that the Notch signal is controlled by the spatiotemporal production and maintenance of Notch receptors and ligands on the cell surface, type of ligand binding to the receptor, glycosylation of the EGF domain that influences ligand-receptor binding, and the amount and stability of NICD [21, 22]. In addition, Notch gene mutations reflecting context-specific selective pressure altered Notch function in various cancers, although mutational activation of Notch is rare-to-absent in PADC [23, 24].

The KLF family transcription factors were demonstrated to regulate Notch signals. *Notch-1, DLL4* and *Hes 1* were direct transcriptional targets of KLF4 in promoting angiogenesis [25]. KLF9 suppressed *Notch-1* transcription in glioblastoma multiforme derived from neurospheres [26]. In this study, we found that KLF10 transcriptionally suppressed *Notch-3* and *-4* and other Notch signaling molecules. Whether KLF family members can share common promoter binding sites on Notch signal targets and regulate the balance between stemness and differentiation in a coordinated manner warrants further exploration.

Given KLF10 is an early response gene of TGFβ, there is also an interplay between Notch and TGFβ signals. In the presence of CSL and NICD, Smad 3 could be recruited to CSL (CBF-1/RBP-JK, Su(H), Lag-1)-binding sites on DNA [27]. KLF10 might also contribute to Smad 3 recruitment in regulating Notch signaling because the levels of phospho-Smad 3 were significantly higher in the tissue of KLF10 L/L mice [28]. Previous studies, including our own, have revealed that KLF10 transcriptionally suppressed Sirtuin 6 and Slug to modulate EMT in PDAC [6, 7]. KLF10 might also modulate stem cell phenotypes of PDAC through EMT and metabolic reprogramming [29, 30].

The Notch signaling pathway exerts both oncogenic and tumor suppressive functions, depending on the cellular context [31]. Recent studies have revealed that real-time Notch signaling dynamics represent another critical component of the pathway function [32]. The conserved Notch target genes responded differently to the level changes of NICD. Sustained or pulsatile Notch activation, NICD dimerization and chromatin opening initiate different gene expression [33, 34]. We found that ELF3 and KLF10 exerted positive and negative effects on *Notch-3* and *Notch-4* gene transcription, respectively. ELF3, playing an important oncogenic role in several tumors including PDAC, was reported to positively regulate *Notch-3* gene transcription and modulate tumor-initiating cells in K-Ras-mediated lung adenocarcinoma [17]. Furthermore, ELF3 showed an upregulated expression in PDAC and was associated with EMT and poor prognosis [35]. Levels of Notch-3 and -4, which affect the dynamics of the Notch signal, may be adjusted by modulating the ELF3/KLF10 level. Transcription factors including E26 transformation specific (ETS) family for binding serine 68 resides within the pointed domain of ELF3 [17] or epigenetic regulation of KLF10 by interacting with corepressor such as histone deacetylases may also contribute to the Notch signal regulated by ELF3/KLF10.

In contrast to Notch-1 and -2, which were expressed widely in many tissues, Notch-3 was found most abundantly in vascular smooth muscle and pericytes, while Notch-4 was highly expressed on endothelial cells [36]. The levels of Notch-1 and -3 were increased in PDAC tissues [37]. Different Notch receptors have discrete functions in PDAC due to the unique downstream targets and expression patterns of the receptors. In mice with oncogenic K-Ras, the deletion of both *Notch-1* alleles accelerated PanIN progression; whereas the deletion of *Notch-2* prolonged survival and delayed PDAC development [31, 38, 39]. The expression of Notch-3 and -4 in PDAC has been correlated with malignant phenotypes and drug resistance [40–43]. Notch-3 was reported to inhibit signaling from the Notch-1 by competing for access to RBP-JK and common coactivator [44]. When expressed in cis, the extracellular domain of Notch-4 also inhibited Notch-1 signaling [45]. The opposite effect of the Notch receptors’ signal warrants caution when using a global Notch inhibition approach for treatment purposes.

In addition to γ-secretase inhibitor, we used evodiamine to suppress Notch-3 expression. Evodiamine, an alkaloid derived from *Euodiae Fructus,* has low toxicity and suppresses Notch-3 by activating DNA methyltransferase 3A induced Notch-3 methylation in non-small-cell lung cancer [12]. Despite its diverse biological effects, we found that evodiamine minimally changed the expression levels of Notch-1 and -4 in PDAC cells. In contrast to some reports of evodiamine’s hepatotoxicity and cardiotoxicity [46], the significant inhibitory effect of evodiamine on tumor size of Panc-1-pLKO-shKLF10 did not result in substantial body weight loss of the mice. Recent studies have also demonstrated the therapeutic efficacy of Notch-3-inhibiting antibodies and Notch-3-targeted antibody–drug conjugate in multiple murine cancer models [47, 48]. Because KLF10 might modulate EMT and metabolic reprogramming to regulate cancer stemness, we combined evodiamine with metformin, which elevated KLF10 expression by modulating AMPK phosphorylation [8], in the orthotopic murine model of PDAC. The combination of evodiamine and metformin therapy achieved significant tumor growth delay without obvious toxicity in mice implanted with Panc-1-pLKO-shKLF10 cells.

In conclusion, we demonstrated a novel signaling pathway of stem cell regulation in PDAC with KLF10 deficiency. We showed that KLF10 competes with ELF3 to bind to *Notch-3* and *Notch-4* genes promoters for transcription regulation. Loss of KLF10, observed in two-thirds of PDAC patients, favored Notch-3 and-4 expression and the further Notch signal transduction, resulting in the development of stem cell phenotype and tumorigenesis in PDAC. Elevating KLF10 expression and suppressing Notch signaling, either genetically or pharmacologically, ameliorated the malignant progression of PDAC with KLF10 deficiency.

## Limitations

To fully exploit the therapeutic potential of our findings, additional work is needed to decipher the KLF10-modulated Notch signaling pathway molecules beyond Notch-3 and -4. The interactions between KLF10 family members and the Notch signaling pathways should also be explored to find potential counterbalances and dynamics in modulating stem cell phenotypes. We used metformin, DAPT, and evodiamine in the murine tumor model to demonstrate that KLF10, the Notch signaling pathway and Notch-3 are potential therapeutic targets in PDAC with KLF10 deficiency. The development of a KLF10 inducer and Notch signal inhibitor with high specificity and low toxicity is warranted to validate the importance of the KLF10/Notch signaling pathways in PDAC.

## Conclusions

A spontaneous murine model of PDAC with additional KLF10 depletion reveals accelerated malignant progression and identifies a mechanism of cancer stem cell phenotype involving Notch signaling activation, suggesting a potential therapeutic target.

## Supplementary Information

**Additional file 1: Figure S1.** Representative colony formation of primary murine PDAC cells from KPCmice as indicated.quantitative bar graphs of mean ± SE from cumulated data of three independent experiments. *p < 0.05,representative flow cytometry of CD133 and CD326 on murine PDAC cell lines from KPC and KKPC mice as indicated.quantitative bar graphs of mean ± SE from cumulated data of three independent experiments from each cell lines developed from KPCor KKPCmice as indicated. *p < 0.05,representative sphere formation of MiaPaCa cells of vector controlor with conditional KLF10 overexpressionwithout or with doxycyclinetreatment as indicated. Original magnification 100× and 400× of upper and lower panel, respectively.immunofluorescence stain of CD44on MiaPaCa-pLVX or MiaPaCa-pLVX-KLF10 without or with Dox treatment as indicated. DAPI was used for nuclei stain.quantitative bar graphs of mean ± SE from cumulated data of three independent experiments from MiaPaCa-pLVXor MiaPaCa-pLVX-KLF10 cells withoutor withDox treatment as indicated. *p < 0.05.

**Additional file 2: Figure S2.** Representative immunofluorescence stain of fluorescence conjugated signal molecules as indicated on spheres of Panc-1-pLKO and Panc-1-pLKP-shKLF10. DAPIwas used for nuclei staining. Original magnification 400×.Representative immunofluorescence stain of KLF10, Notch-3 and -4 as indicated on spheres of MiaPaCa-pLVX or MiaPaCa-pLVX-KLF10 without or with Dox treatment. DAPIwas used for nuclei staining. Original magnification 400×.Representative immunoblots of Notch signal molecules expression of MiaPaCa-pLVX or MiaPaCa-pLVX-KLF10 without or with Dox treatment. β-actin was used as internal control.

**Additional file 3: Figure S3.** Primer design ofNotch3/4 promoters for ChIP assay. Primer design for cloning ofNotch-3 andNotch-4 promoters of various deletions.Primer design for ELF3 and mutant S68ELF3.Representative GEO databases of correlation between ELF 3 versus Notch-3, Notch-4, and KLF10transcripts levels in pancreatic adenocarcinoma.

**Additional file 4: Figure S4.** Representative sphere formation of Panc-1-pLKO and Panc-1-pLKO-shKLF10 without or with Notch-3 or Notch-4 depletion as indicated. Original magnification 100×Representative sphere formation of Panc-1-pLKO-shKLF10 without or with 5 µM DAPT treatment. Original magnification 100×.

**Additional file 5: Figure S5.** Body weight of mice implanted orthotopically with Panc-1-pLKO-shKLF10 and treated with PBS, metformin, evodiamineor concomitant metformin and evodiamine. Each point represents mean ± SE from cumulated data of at least 5 mice.

**Additional file 6: Table S1.** 10 pathway QPCR primer sequence.Notch pathway QPCR primer sequence.

**Additional file 7.** Additional materials and methods.

## Data Availability

The datasets used and/or analyzed during the current study are available from the corresponding author on reasonable request. The microarray data have been deposited in GEO under accession number GSE218172. Any additional information required to reanalyze the data reported in this paper is available from the lead contact upon request.

## Abbreviations

KLF10: Krüppel-like factor 10
PDAC: Pancreatic adenocarcinoma
KC mice: LSL: Kras^G12D^; Pdx1-Cre mice
KLF10L/L: KLF10^Loxp/Loxp^ mice
KPC cells: Primary cancer cell lines from Pdx-1Cre, LSL-Kras^G12D^ and p53^Loxp/Loxp^ mice
KKPC cells: Primary cancer cell lines from Pdx-1Cre, LSL-Kras^G12D^ p53^Loxp/Loxp^ and KLF10^Loxp/Loxp^ mice
NOD/SCIDmice: Nonobese diabetic /severe combined immunodeficient mice
Panc-1-pLKO-shKLF10: Stably depleting KLF10 in Panc-1 cells
MiaPaCa-pLVX-KLF10: Stable doxycycline inducible clones of KLF10 insert under the TRE3G promoter in MiaPaCa cells
Panc-1-Luc: Panc-1 cells labeled with firefly luciferase plasmid vector
ELF3: E74-like ETS transcription factor 3
AMPK: Adenosine monophosphate-activated protein kinase
NICD: Notch intracellular domains
TIEG1: TGFβ-inducible early gene-1
EMT: Epithelial–mesenchymal transition
SP-1: Specific protein-1
CHIP: Chromatin immunoprecipitation
qRT-PCR: Quantitative real-time polymerase chain reaction
IPA: Ingenuity pathway analysis
GSEA: Gene set enrichment analysis
PBS: Phosphate-buffered saline
IVIS: In vivo imaging system
EI score: Extent and intensity score of immunohistochemical stain
Compound C: Dorsomorphin
DAPT: *N*-[*N*-(3,5-Difluorophenacetyl-l-alanyl)]-(*S*)-phenylglycine *t*-butyl ester
