# Supplementary figures and images for: Krüppel-like factor 10 modulates stem cell phenotypes of pancreatic adenocarcinoma by transcriptionally regulating notch receptors
Source: J Biomed Sci. 2023 Jun 12;30:39. doi: 10.1186/s12929-023-00937-z (PMC10258947; doi:10.1186/s12929-023-00937-z)

**Figure S1**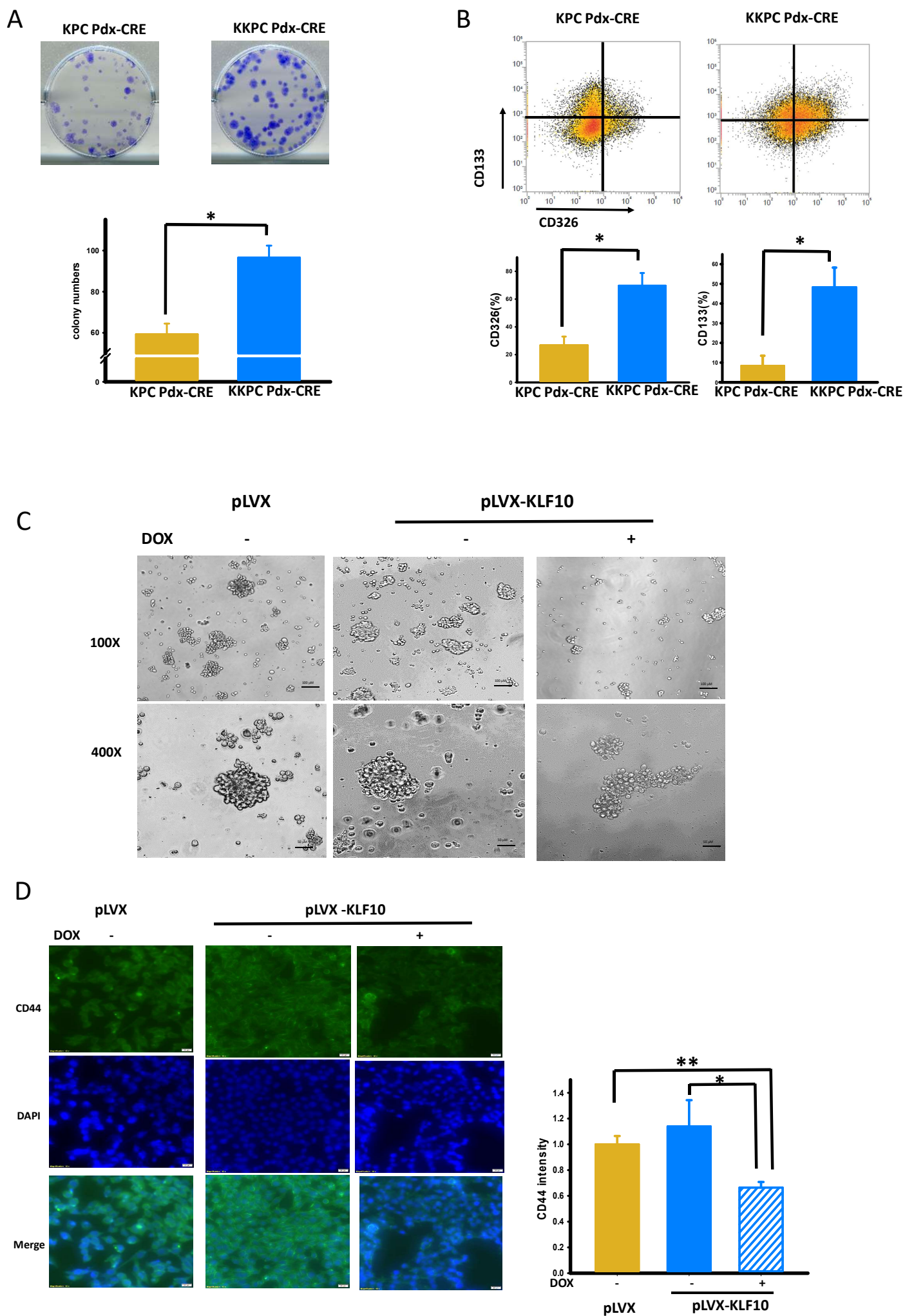

Supplement: Supplementary file 1 — Additional file 1: Figure S1. Representative colony formation of primary murine PDAC cells from KPCmice as indicated.quantitative bar graphs of mean ± SE from cumulated data of three independent experiments. *p < 0.05,representative flow cytometry of CD133 and CD326 on murine PDAC cell lines from KPC and KKPC mice as indicated.quantitative bar graphs of mean ± SE from cumulated data of three independent experiments from each cell lines developed from KPCor KKPCmice as indicated. *p < 0.05,representative sphere formation of MiaPaCa cells of vector controlor with conditional KLF10 overexpressionwithout or with doxycyclinetreatment as indicated. Original magnification 100× and 400× of upper and lower panel, respectively.immunofluorescence stain of CD44on MiaPaCa-pLVX or MiaPaCa-pLVX-KLF10 without or with Dox treatment as indicated. DAPI was used for nuclei stain.quantitative bar graphs of mean ± SE from cumulated data of three independent experiments from MiaPaCa-pLVXor MiaPaCa-pLVX-KLF10 cells withoutor withDox treatment as indicated. *p < 0.05. [file 12929_2023_937_MOESM1_ESM.pdf]

Figur e S2

A

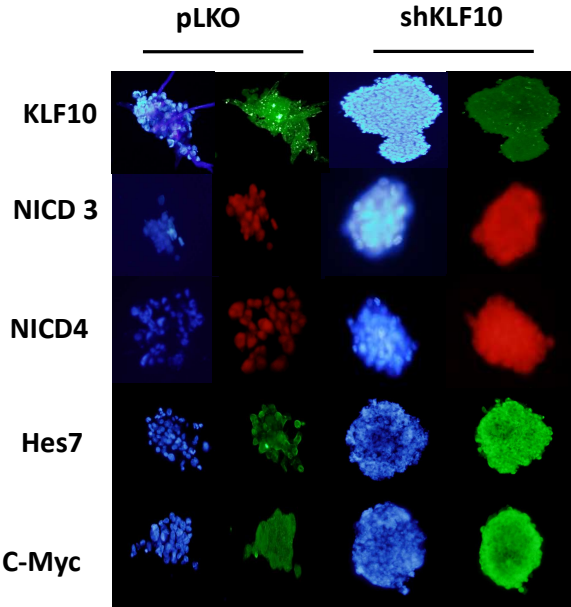

B

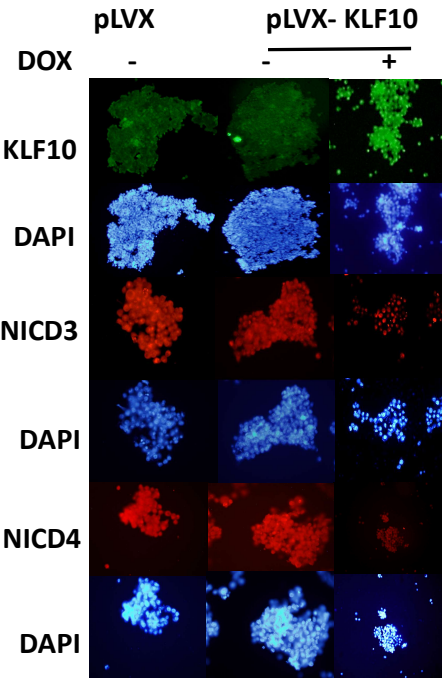

C

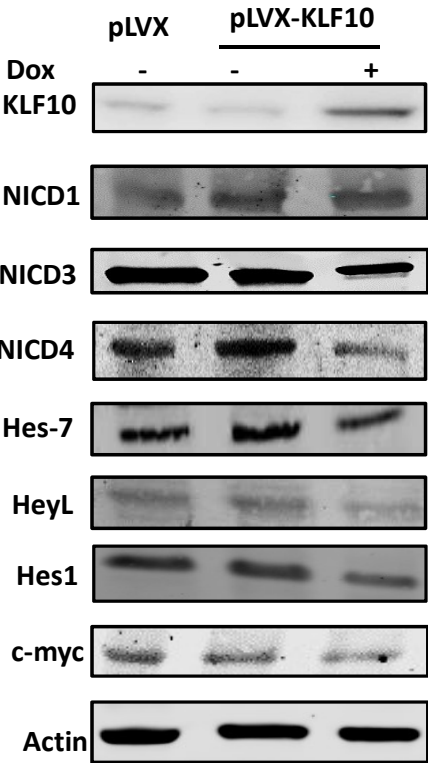

Supplement: Supplementary file 2 — Additional file 2: Figure S2. Representative immunofluorescence stain of fluorescence conjugated signal molecules as indicated on spheres of Panc-1-pLKO and Panc-1-pLKP-shKLF10. DAPIwas used for nuclei staining. Original magnification 400×.Representative immunofluorescence stain of KLF10, Notch-3 and -4 as indicated on spheres of MiaPaCa-pLVX or MiaPaCa-pLVX-KLF10 without or with Dox treatment. DAPIwas used for nuclei staining. Original magnification 400×.Representative immunoblots of Notch signal molecules expression of MiaPaCa-pLVX or MiaPaCa-pLVX-KLF10 without or with Dox treatment. β-actin was used as internal control. [file 12929_2023_937_MOESM2_ESM.pdf]

Figure S4

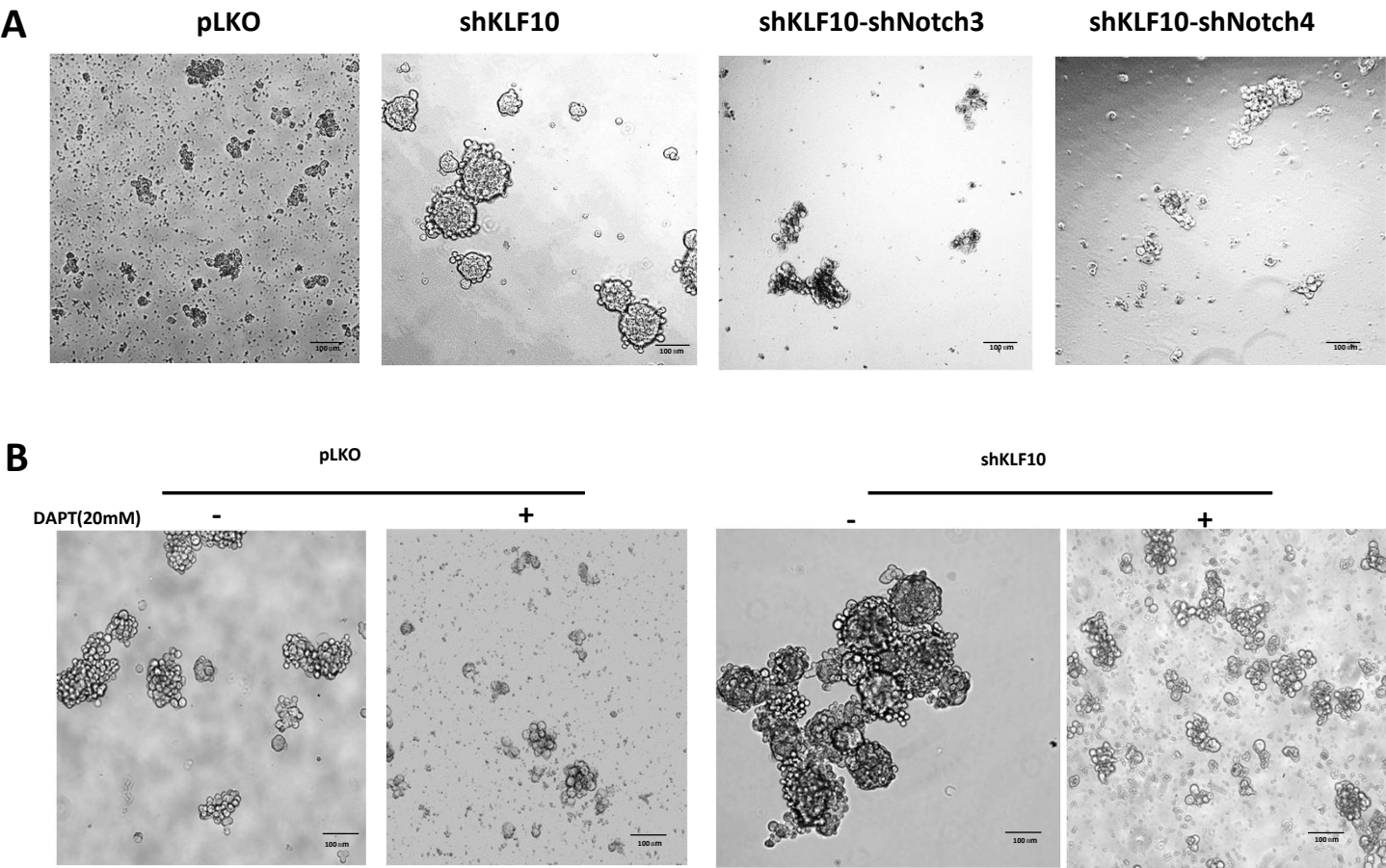

Supplement: Supplementary file 4 — Additional file 4: Figure S4. Representative sphere formation of Panc-1-pLKO and Panc-1-pLKO-shKLF10 without or with Notch-3 or Notch-4 depletion as indicated. Original magnification 100×Representative sphere formation of Panc-1-pLKO-shKLF10 without or with 5 µM DAPT treatment. Original magnification 100×. [file 12929_2023_937_MOESM4_ESM.pdf]

Figure S5

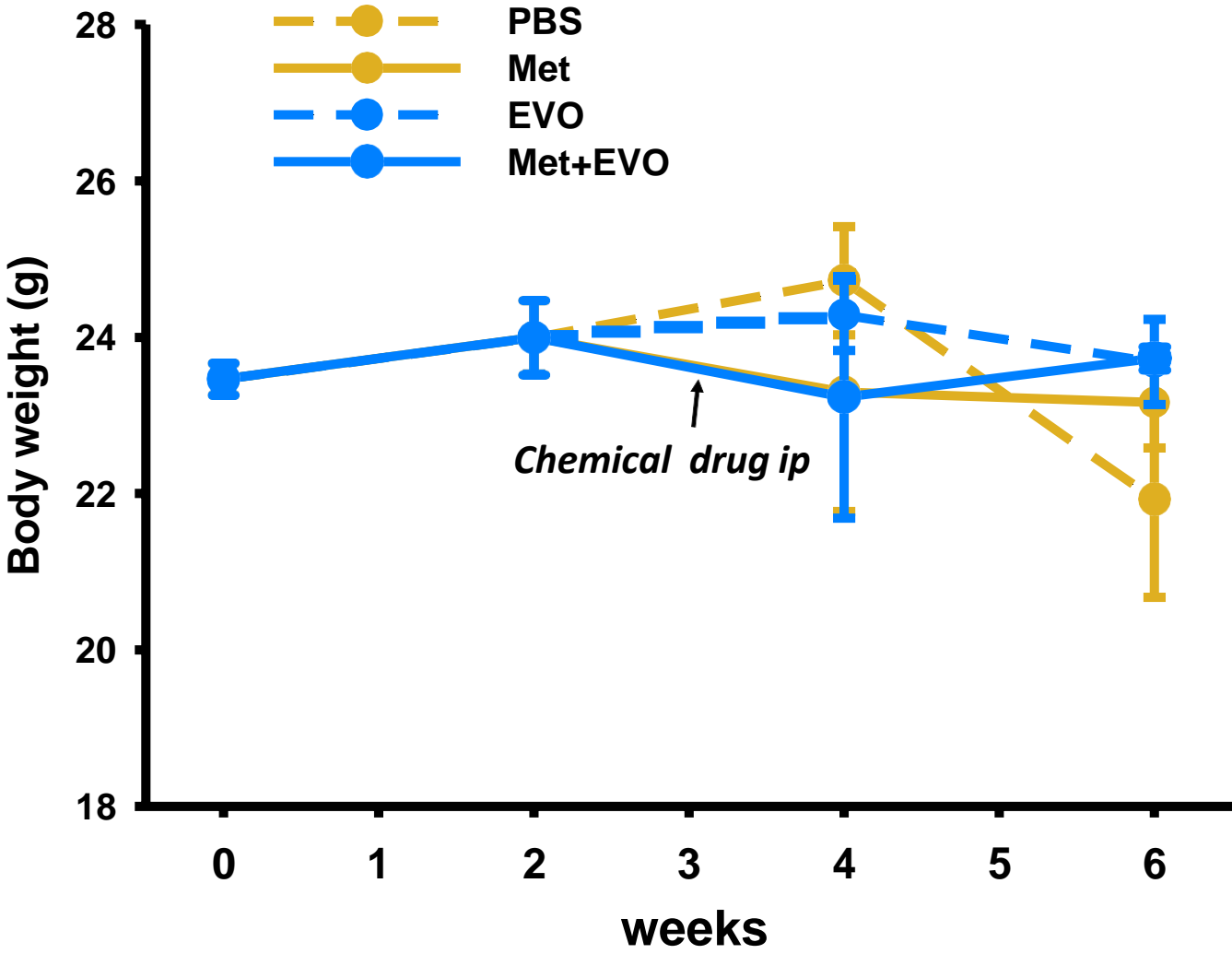

Supplement: Supplementary file 5 — Additional file 5: Figure S5. Body weight of mice implanted orthotopically with Panc-1-pLKO-shKLF10 and treated with PBS, metformin, evodiamineor concomitant metformin and evodiamine. Each point represents mean ± SE from cumulated data of at least 5 mice. [file 12929_2023_937_MOESM5_ESM.pdf]
