# Supplementary material for: Krüppel-like factor 10 modulates stem cell phenotypes of pancreatic adenocarcinoma by transcriptionally regulating notch receptors
Source: J Biomed Sci. 2023 Jun 12;30:39. doi: 10.1186/s12929-023-00937-z (PMC10258947; doi:10.1186/s12929-023-00937-z)
Supplement: Supplementary file 3 — Additional file 3: Figure S3. Primer design ofNotch3/4 promoters for ChIP assay. Primer design for cloning ofNotch-3 andNotch-4 promoters of various deletions.Primer design for ELF3 and mutant S68ELF3.Representative GEO databases of correlation between ELF 3 versus Notch-3, Notch-4, and KLF10transcripts levels in pancreatic adenocarcinoma. [file 12929_2023_937_MOESM3_ESM.pdf]

Figure S3

A. Notch3 & Notch4 ChIP assay Promoter Primers

Notch3
Forward: TCACAGAGGAAGTGGGTTGC
Reveres: CAGCCTCAGACCTCAGACA

Notch4
Forward: CCCCAAAGTTGTCCTGGGTT
Reverse: TCCTTGGGATGCAGGGAATG.

B. Notch3 Promoter Cloning Primers

-986
Forward: ***Kpn1*** GG***GGTACC*** TCACAGAGGAAGTGGGTTGC
Reveres: ***Xho1*** GG ***CTCGAG*** GGGTTCTTGCACTCCCCCTCT

-725
Forward: ***Kpn1*** GG***GGTACC*** GGGTTCTTGCACTCCCCCTCT
Reveres: ***Xho1*** GG ***CTCGAG*** GGGTTCTTGCACTCCCCCTCT

-352
Forward: ***Kpn1*** GG***GGTACC*** CGGCTCTGGGTGTGTACTG
Reveres: ***Xho1*** GG ***CTCGAG*** GGGTTCTTGCACTCCCCCTCT

C. Notch4 Promoter Cloning Primers

-938
Forward: ***Kpn1*** GG***GGTACC*** CCCCAAAGTTGTCCTGGGTT
Reveres: ***Xho1*** GG ***CTCGAG*** CACCTCTGGGTCTGACCACT

-798
Forward: ***Kpn1*** GG***GGTACC*** GGCAGAGATGGAGGAAGTGA
Reveres: ***Xho1*** GG ***CTCGAG*** CACCTCTGGGTCTGACCACT

-325
Forward: ***Kpn1*** GG***GGTACC*** GGTTCCGAGATTCCTTCTCC
Reveres: ***Xho1*** GG ***CTCGAG*** CACCTCTGGGTCTGACCACT

D. ELF3 cloning & KLF10 cloning

a.ELF3 NM_001114309.2 ***cds*** synthesis subcloned in pcDNA3.1(+)

b.ELF3 Mutagenesis Primers subcloned in pcDNA3.1(+)
S68(A) mutant:
Forward: GGAACAGCCCCAGTTCTGG***GCG***AAGACGCAGGTTCTGGACTGG
Reverse: CCAGTCCAGAACCTGCGTCTT***CGC***CCAGAACTGGGGCTGTTCC

c. KLF10 NM_005655.4 subcloned pcDNA3

E.

ELF3 vs Notch3	NO. of sample	Correlation
GSE71729	158 (pancreatic cancer)	0.13
GSE56560	35	0.18
GSE57495	63	0.20
GSE17891	47 (Human pancreatic cancer)	0.93

ELF3 vs Notch4	NO. of sample	Correlation
GSE71729	158 (pancreatic cancer)	0.08
GSE56560	35	0.30
GSE17891	47 (Human pancreatic cancer)	0.90

ELF3 vs KLF10	NO. of sample	Correlation
GSE22780	16	-0.16
GSE28735	90	-0.21
GSE32676	42	-0.11
GSE46234	8	-0.13
GSE16515	52	-0.22
GSE19650	22	-0.71
GSE101448	43	-0.15
