# Supplementary material for: Krüppel-like factor 10 modulates stem cell phenotypes of pancreatic adenocarcinoma by transcriptionally regulating notch receptors
Source: J Biomed Sci. 2023 Jun 12;30:39. doi: 10.1186/s12929-023-00937-z (PMC10258947; doi:10.1186/s12929-023-00937-z)
Supplement: Supplementary file 6 — Additional file 6: Table S1. 10 pathway QPCR primer sequence.Notch pathway QPCR primer sequence. [file 12929_2023_937_MOESM6_ESM.pdf]

Table S1

A. 10 pathway QPCR primer sequence

Gene name	*Forward*	*Reverse*
Myc	*CACCTTGTAGCACGTCCTG*	*GACTCCCCAAGATGTGGTGG*
PAX6	*TGGGCAGGTATTACGAGACTG*	*ACTCCCGCTTATACTGGGCTA*
MEF2	*CGTGCTATGTGACTGCGAGAT*	*GCGTCGGTACTTGTCCTCC*
GLI (Hedgehog)	*TCTGCCCCCATTGCCCACTTG*	*TACATAGCCCCCAGCCCATACCTC*
Wnt(TCF/LEF)	*AAACAGGAACATCCCCACAC*	*TCAGAGGCTTCTTAATGTGAGGT*
Oct4	*GTGTTCAGCCAAAAGACCATCT*	*GGCCTGCATGAGGGTTTCT*
KLF4	*CCCACATGAAGCGACTTCCC*	*CAGGTCCAGGAGATCGTTGAA*
Notch(RBP-Jk)	*AACAAATGGAACGCGATGGTT*	*GGCTGTGCAATAGTTCTTTCCTT*
Nanog	*TTTGTGGGCCTGAAGAAAACT*	*AGGGCTGTCCTGAATAAGCAG*
SOX2	*TCCCGTATGAAAGCATCGTGG*	*CCCATTTGGGTAGATCAGGTAAC*

B. Notch pathway QPCR primer sequence

Gene name	*Forward*	*Reverse*
Notch1	GAGGCGTGGCAGACTATGC	CTTGTACTCCGTCAGCGTGA
Notch2	GATCACCCGAATGGCTATGAAT	GGGGTCACAGTTGTCAATGTT
Notch3	CGTGGCTTCTTTCTACTGTGC	CGTTCACCGGATTTGTGTCAC
Notch4	GATGGGCTGGACACCTACAC	CACACGCAGTGAAAGCTACCA
Pen-2	CTGGAGCGAGTGTCCAATGAG	GCGCCAGACATAGCCTTTGAT
DTX2	TGGCTCCTGGACTGCCTAT	GGGTGGTGTAGTTGACAGTGTA
DLL1	GATTCTCCTGATGACCTCGCA	TCCGTAGTAGTGTTCGTCACA
Jag1	GTCCATGCAGAACGTGAACG	GCGGGACTGATACTCCTTGA
Kat2A	CAGGGTGTGCTGAACTTTGTG	TCCAGTAGTTAAGGCAGAGCAA
Hes1	ACGTGCGAGGGCGTTAATAC	GGGGTAGGTCATGGCATTGA
Hes7	CGGGATCGAGCTGAGAATAGG	GCGAACTCCAATATCTCCGCTT
HeyL	GGAAGAAACGCAGAGGGATCA	CAAGCGTCGCAATTCAGAAAG
Maml2	CACAGTGCTATCGTGGAGCG	CTCGTTCATATCGTCCTTCACAG
myc	*CACCTTGTAGCACGTCCTG*	*GACTCCCCAAGATGTGGTGG*
