# Supplementary material for: Krüppel-like factor 10 modulates stem cell phenotypes of pancreatic adenocarcinoma by transcriptionally regulating notch receptors
Source: J Biomed Sci. 2023 Jun 12;30:39. doi: 10.1186/s12929-023-00937-z (PMC10258947; doi:10.1186/s12929-023-00937-z)
Supplement: Supplementary file 7 — Additional file 7. Additional materials and methods. [file 12929_2023_937_MOESM7_ESM.docx]

**Additional Materials and Methods**

**Colony formation**

Four hundred cells per well were seeded onto a six-well plate. After 7-9 days of incubation, medium was removed. Cells were washed with 1 x phosphate-buffered saline (PBS) and fixed with methanol/acetic acid (v/v:3/1) for 5 min, and were stained with 0.5% crystal violet for 15 min. Colonies ≥ 50 cells were counted using light microscope (Olympus IX73, Tokyo, Japan) by two operators, from which an average was calculated.

**Sphere formation assay**

Cells were plated in ultra-low attachment plates (Corning, Oneonta, NY) at a density of 5x10^4^ cells/well in 3 ml of PSGro hESC/iPSC growth medium (VHM01, Empowering StemRD, Burlingame, CA) for 6-10 days when spheres reached a diameter of 50μm. The formed spheres were counted by inverted microscopy. Sphere formation efficiency was calculated to be sphere/input cells x 100%.

**In vivo imaging system**

A dose of 125 mg/kg of body weight luciferin (L8220, Biosynth Carbosynth LLC, San Diego, CA), the substrate of luciferase, was injected intraperitoneally into mice. The mice were then anesthetized and placed in supine position on the imaging stage. Images were collected from 10 - 30 min after luciferin injection using *in vivo* imaging system (IVIS; Xenogen, Hopkinton, MA), and the photons emitted from the tumor were quantified using Living Image Software (Xenogen). Tumor necrosis with low signal intensity was excluded by histologic examination.

**Immunofluorescence staining**

For immunofluorescence staining, cells or spheres were fixed with 3.7% paraformaldehyde for 5 min followed by fixing buffer (methanol/acetic acid: 3/1) on ice for 10 min. Slides were blocked with 5% BSA in PBS at room temperature for 30 min and incubated with antibodies including KLF10 (1:400, LTK BioLaboratories), Notch-3 (1:500, ab23426, Abcam) , Notch-4 (1:200, ab199295, Abcam), Hes 7 (1:100, E-ab-18076, Elabscience, Houston, Texas), c-Myc (1:1000 , E-ab-30975 Elabscience) and CD47 (1:200,GTX63166, GeneTex, Irvine, CA) at 4°C overnight. After repeated washings, cells were incubated with Alexa Fluor 488 (Cat#B40953, Invitrogen, Carlsbad, CA) or Alexa Fluor 594 (Cat#A11012, Invitrogen) -conjugated secondary antibody for fluorescent detection, counterstained with 4',6-diamidino-2-phenylindole (DAPI) and quantitative analysis by Image J (National Institutes of Health, Bethesda, MA).

**Patient specimens and statistics**

Pancreatic tumor specimens were obtained from 110 of 147 patients with curatively resected PDAC enrolled in a randomized phase III clinical trial (ClinicalTrials.gov. identifier: NCT 00994721) (14). Eligible patients were 20 to 75 years old, with adequate organ function and serum CA19-9 levels less than 2.5 x the institutional upper limit. Only 105 specimens were optimal for evaluating KLF10 immunostaining (ClinicalTrials.gov. identifier: NCT01666184). The tissue slides were examined independently by two observers (S.L.P. and C.C.) who were masked to both the clinical and pathological data. Immunostaining was assessed regarding cellular localization, intensity and distribution. The expression of biomarkers was quantified using a visual grading system based on the extent of staining (percentage of positive tumor cells graded on a scale of 0 to 3: 0, 0%; 1, 1% - 30%; 2, 31% - 60%; and 3, > 60%) and the intensity of staining (graded on a scale of 0 to 3: 0, no staining; 1, weak staining; 2: moderate staining; and 3, strong staining). The combination of the extent (E) and intensity (I) of staining was obtained by calculating E × I to determine the extent-intensity (EI), which varied from 0 to 9. The mean EI score was calculated for each pancreatic cancer specimen. For the statistical analysis of KLF10, EI scores of 0-1 were considered low expression, and EI scores > 1 were considered high expression. The correlations of EI scores between KLF10 and Notch-3/-4 were analyzed in 31, and 29 patients, respectively, from the cohort mentioned above.

SPSS (v22.0, IBM, Armonk, NY), Sigmaplot (v12.5, SYSTAT, Plano, TX) and the GraphPad Prism software (v8.03, Dotmatics, Boston ,MA) were used for statistical analyses of group comparisons of normally distributed data by the independent Student’s *t*-test or one-way analysis of variance. Pearson’s correlation analysis was used to determine the correlation between the expression of two molecules. Kaplan-Meier analysis and the log-rank test were used to analyze overall survival and distant metastasis-free survival. Statistical differences were considered significant at *p* < 0.05.
